# CDG Therapies: From Bench to Bedside

**DOI:** 10.3390/ijms19051304

**Published:** 2018-04-27

**Authors:** Sandra Brasil, Carlota Pascoal, Rita Francisco, Dorinda Marques-da-Silva, Giuseppina Andreotti, Paula A. Videira, Eva Morava, Jaak Jaeken, Vanessa dos Reis Ferreira

**Affiliations:** 1 Portuguese Association for Congenital Disorders of Glycosylation (CDG), Departamento Ciências da Vida, Faculdade de Ciências e Tecnologia, Universidade NOVA de Lisboa, 2820-287 Lisboa, Portugal; s.arduim@gmail.com (S.B.); carlotapascoal@gmail.com (C.P.); rab.francisco@campus.fct.unl.pt (R.F.); dorindams@gmail.com (D.M.-d.-S.); p.videira@fct.unl.pt (P.A.V.); 2 Professionals and Patient Associations International Network (CDG & Allies—PPAIN), Departamento Ciências da Vida, Faculdade de Ciências e Tecnologia, Universidade NOVA de Lisboa, 2820-287 Lisboa, Portugal; emoravakozicz@tulane.edu; 3 Research Unit on Applied Molecular Biosciences (UCIBIO), Departamento Ciências da Vida, Faculdade de Ciências e Tecnologia, Universidade NOVA de Lisboa, 2829-516 Lisboa, Portugal; 4 Istituto di Chimica Biomolecolare-Consiglio Nazionale delle Ricerche (CNR), 80078 Pozzuoli, Italy; gandreotti@icb.cnr.it; 5 Department of Clinical Genomics, Mayo Clinic, Rochester, MN 55905, USA; 6 Center for Metabolic Diseases, Universitaire Ziekenhuizen (UZ) and Katholieke Universiteit (KU) Leuven, 3000 Leuven, Belgium

**Keywords:** animal models, biomarkers, clinical trials, congenital disorders of glycosylation (CDG), diagnosis, dietary supplementation, mannose, galactose, pharmacological chaperones, therapy

## Abstract

Congenital disorders of glycosylation (CDG) are a group of genetic disorders that affect protein and lipid glycosylation and glycosylphosphatidylinositol synthesis. More than 100 different disorders have been reported and the number is rapidly increasing. Since glycosylation is an essential post-translational process, patients present a large range of symptoms and variable phenotypes, from very mild to extremely severe. Only for few CDG, potentially curative therapies are being used, including dietary supplementation (e.g., galactose for PGM1-CDG, fucose for SLC35C1-CDG, Mn^2+^ for TMEM165-CDG or mannose for MPI-CDG) and organ transplantation (e.g., liver for MPI-CDG and heart for DOLK-CDG). However, for the majority of patients, only symptomatic and preventive treatments are in use. This constitutes a burden for patients, care-givers and ultimately the healthcare system. Innovative diagnostic approaches, in vitro and in vivo models and novel biomarkers have been developed that can lead to novel therapeutic avenues aiming to ameliorate the patients’ symptoms and lives. This review summarizes the advances in therapeutic approaches for CDG.

## 1. Introduction

Congenital disorders of glycosylation (CDG) represent a group of genetic disorders with impaired synthesis and attachment of glycans to glycoproteins and glycolipids, and impaired synthesis of glycosylphosphatidylinositol (GPI) [1]. The more than 100 known CDG can be divided into: (a) protein *N*-glycosylation defects; (b) protein *O*-glycosylation defects; (c) glycolipid and GPI anchor synthesis defects and (d) multiple glycosylation pathways and other pathway defects [2,3,4,5]. The last category encompasses defects affecting vesicular transport, nucleotide-sugar transport or *O*-mannosylation, among others. The subcellular location of the defect can also be used as a complementary classification [6]. Among the different glycosylation defects, protein *N*-glycosylation defects are the most common. In *N*-glycosylation a glycan is first assembled as precursor attached to dolichol phosphate at the cytoplasmic side of the endoplasmic reticulum (ER) and then transferred to the amide nitrogen of an asparagine in the consensus glycosylation site (Asn-X-Ser/Thr) of the growing polypeptide in the lumen of the ER. Glycan processing (remodeling) takes place in the ER and Golgi apparatus giving rise to a complex, mature glycan [3,7]. *O*-glycosylation occurs in the Golgi apparatus and no lipid-linked intermediates are involved. In contrast to *N*-glycosylation, the process consists only in the sequential addition of monosacharides without remodeling and the most common structure is obtained by the addition of an *N*-acetylgalactosamine (GalNAc) residue to the hydroxyl group of a serine or threonine [3,7]. Defects in the synthesis of O-linked *N*-acetylglucosamine (GlcNAc), galactose (Gal), mannose (Man), glucose (Glc) and fucose (Fuc) glycans have also been described [8].

Glycosylation is essential for a variety of biological processes and thus CDG patients present highly heterogeneous clinical phenotypes that can range from mild to severe and involve single or in most cases, multiple organs [4,7]. Neurological symptoms include cognitive impairment, epilepsy, hypotonia, ataxia and polyneuropathy. Other symptoms are ophthalmological, skeletal, cardiac, hepatic, hematological and endocrinological [3,4,9,10]. Serum transferrin isoelectrofocusing is still the screening method of choice. An abnormal result is followed by CDG panel/whole-exome (WES)/whole genome (WGS) sequencing, among other strategies, to identify the involved gene [1,11,12]. 

Transferrin glycosylation patterns were also the basis for the first CDG classification (e.g., CDG-I or CDG-II). However, this nomenclature comprises only *N*-glycosylation deficiencies leaving out others, such as *O*-glycosylation defects (e.g., EOGT-CDG, POGLUT1-CDG or B4GALT7-CDG) [13]. Hence, a new, clear and informative nomenclature was implemented based on the name of the affected gene (not in italics) followed by “-CDG” [13]. The growing number of known CDG has stimulated research into their pathophysiological mechanisms in order to achieve better disease management and ultimately an efficient treatment. Several in vitro and in vivo disease models have been generated allowing the identification of important disease hallmarks, such as novel biomarkers and the screening and testing of possible therapeutic approaches. As a result, advances in treatment options have been made for some CDG [14,15,16] but the majority of CDG still remain without effective treatment. 

In order to collect information about CDG for which some form of treatment already exists (in pre-clinical research, clinical development, compassionate use or approved stage) we performed an extensive search and review of the literature on the following CDG: ALG1-CDG, ALG6-CDG, ALG13-CDG, ATP6VAP1-CDG, CAD-CDG, CCDC115-CDG, DOLK-CDG, GNE-CDG, ISPD-CDG, MAGT1-CDG, MPI-CDG, NANS-CDG, PGM1-CDG, PGM3-CDG, PIGM-CDG, PIGA-CDG, PIGO-CDG, PMM2-CDG SL39A8-CDG, SLC35C1-CDG, SLC35A2-CDG, SDR5A3-CDG, TMEM165-CDG and TMEM199-CDG. 

The information collected was organized in different sections namely disease models, biomarkers, dietary supplementation therapies, other therapeutic options and clinical trials registered for CDG, therefore following and reporting on therapy development from bench to bedside. 

Hence, this review aims to facilitate further research and therapy development. This should help clinicians and families in disease management and treatment selection.

## 2. Disease Models

In vitro models (e.g., Chinese hamster cell lines or HeLa cell lines) have been extremely useful in CDG to identify biological processes, investigate underlying molecular mechanisms [17,18,19,20,21], categorize new mutations as disease-causing and assess protein functionality [22,23,24,25,26,27,28,29,30,31,32,33,34,35,36]. In vitro models are also often used to study the effect of disrupted enzyme activity due to a pathogenic genetic variation [33,35,37,38,39]. In line with this, they are used as heterologous expression systems for recombinant proteins, allowing the study of the impact of different variants on protein localization and activity, and also their correlation with phenotype severity [30,38,40,41,42,43,44]. Other important in vitro models are patient-derived cells, such as fibroblasts, leukocytes or muscle cells which have been used to test biochemical and cellular features as well as responses to potential treatments. [23,27,36,40,43,45,46,47,48,49,50,51,52,53,54,55,56,57,58,59,60,61,62,63,64,65,66,67,68,69,70,71]

Nevertheless in vitro models present limitations that can only be circumvented by the analysis of a model organism such as, the effect of glycosylation defects in embryonic development or the severe manifestations caused by Man supplementation on an MPI-CDG mouse model [16,72]. Moreover, pre-clinical research data concerning absorption, distribution, metabolism, excretion and toxicity (ADME-Tox) parameter obtain using in vivo models, is essential for further clinical research.

The development of new techniques such as Clustered Regularly Interspaced Short Palindromic Repeats (CRISPR)/Cas9 have made in vivo gene engineering a more accessible and affordable task [73]. One of the major concerns in producing a model organism is the recapitulation of the expected phenotype. Complete deletion of genes involved in glycosylation has caused embryonic or neonatal lethality [74,75] or no disease phenotype [76], while the generation of hypomorphic models has yielded similar results [77]. 

Thus, animal models that replicate the phenotypes of patients are necessary to assess safety and efficacy of new therapeutic approaches for CDG. Despite the difficulties encountered in generating efficient in vivo models, advances have been made that have allowed a better understanding of the pathophysiological mechanisms underlying CDG and also the pre-clinical testing of compounds such as sialic acid (SA), [78,79], *N*-acetylmannosamine (ManNAc) [78,80,81,82], peracetylated *N*-acetylmannosamine (Ac_4_ManNAc) [83], and 6′-sialyllactose [84] for GNE-CDG. 

Several species and engineering techniques have been used in attempts to generate suitable disease models for CDG (Table 1). Several other possible mouse models for CDG are under construction (e.g., C57BL/6NJ-C57BL/6NJ-*Alg8*/Mmjax, C57BL/6NJ-C57BL/6NJ-*Alg9*/Mmjax or 024331-B6N(Cg)-*Alg2*/J) by the Knockout Mouse Project (KOMP) as part of the International Mouse Phenotyping Consortium effort to generate and characterize 5000 targeted knockout mouse lines [85,86]. 

## 3. Biomarkers

Biomarkers are essential for diagnostics, assessment of disease progression and ultimately quantification of the effect of therapeutic approaches. Transferrin glycosylation has been the main biomarker for CDG screening and therapy monitoring. The analysis of its *N*-glycosylation profile by different techniques (reviewed in [4]) is widely used. Nevertheless, the existence of CDG with normal transferrin glycosylation, the normalization of glycosylation patterns with age in several CDG and charge altering transferrin variants, stress the need for complementary biomarkers [154,155]. Currently, clinical CDG screening for *N*-glycosylation disorders relies mainly on isoelectric focusing (IEF) of serum transferrin (TF) and α_1_-antitrypsin, whereas for some *O*-glycosylation disorders it relies on apolipoprotein C-III (apoC-III) IEF (Table 2) [7,155,156,157]. *N*-glycan profile analysis by mass spectrometry (MS) is also used for CDG types for which transferrin profile analysis cannot be used. Plasma glycosylation features from *N*-glycans and intact transferrin used for CDG diagnostics are reviewed in Bakar et al. [8].

Besides these markers used for diagnosis, others have been described in the literature in research or clinical context and are collected in Table 3. 

## 4. Dietary Supplementation Therapies

Various inherited metabolic disorders (IMDs) respond to dietary supplementation and/or restriction strategies [158,159]. In this section, we analyze the effects of supplementation with sugars (e.g., mannose, galactose, fucose), nucleotides (e.g., uridine) and trace elements (e.g., manganese, magnesium) in CDG. 

### 4.1. Defects Located in the Cytosol

#### 4.1.1. Defects in Protein *N*-Glycosylation

##### MPI-CDG

**Figure 1:**
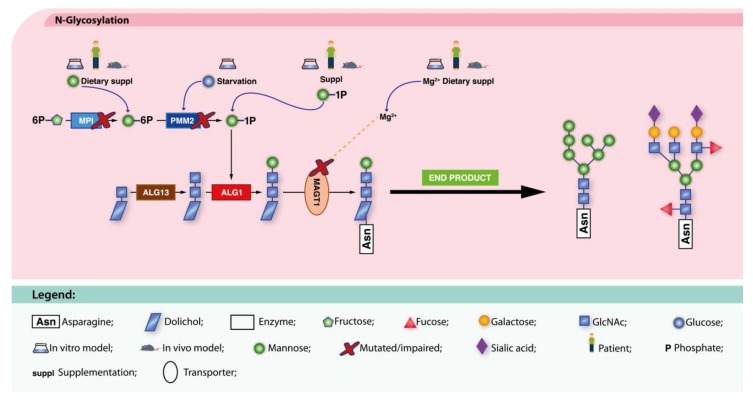
Dietary supplementation approaches under investigation for ALG1-CDG, MPI-CDG, PMM2-CDG and MAGT1-CDG. For MPI-CDG and PMM-CDG, exogenous Man or Man-1-P respectively, is administered (blue arrows). For PMM2-CDG, glucose starvation (blue arrow) was also studied. For ALG1-CDG, Man-1-P supplementation (black arrow) might also represent a promising approach. Mg^2+^ supplementation (blue arrow) is used to correct MAGT1 transporter defect (dotted yellow line). These therapeutic approaches aim to recover the metabolic pathways (black arrows) and ultimately, the normal glycosylation profile (bold black arrow).

MPI-CDG (MIM: 602579) is a disorder of the mannose metabolism due to deficient function of the enzyme mannose-6-phosphate isomerase (MPI, EC 5.3.1.8), responsible for the interconversion of fructose-6-P (Fru-6-P) and mannnose-6-P (Man-6-P) [91]. Man supplementation was first tried in a patient in 1998 [49]. Remarkable biochemical and clinical improvement has been registered in treated MPI-CDG patients, including disappearance of hypoglycemia and gastrointestinal manifestations, normalization of coagulation, improvement of transferrin glycosylation profile, and normal growth (Figure 1). At least 12 MPI-CDG patients have been successfully treated with Man [49,160,161,162,163,164,165,166,167,168,169], however, Man therapy can show side effects that have caused therapy discontinuation in a few patients [165,170]. It also fails to correct overall glycosylation profile [162] and treat hepatic disease [168,171]. This can be due to the liver’s distinct Man requirements [172], or to mannose inability to correct liver disease progression [92,168]. In animal models, contradictory effects of Man supplementation, have been reported regarding embryonic lethality [72,91,92]. These reports sparked the discussion on the possible dangers associated with pre-natal therapy, and on the most effective moment/age to initiate treatment. It also highlighted possible negative long-term effects of continued sugar supplementation. Difference in therapy responses may be explained by disease heterogeneity and severity, route of Man administration (e.g., IV vs. oral), genetic background, and/or age of treatment initiation [162,165,170,173].

Interestingly, high plasma aspartylglucosaminidase (AGA) activity and reduced intercellular adhesion molecule 1 (ICAM-1) expression have been suggested as biomarkers of CDG-I, including MPI-CDG in which AGA and ICAM-1 levels normalized after Man treatment [166,174]. Moreover, Man-mediated elevation of ICAM-1 has been connected to an enhanced immune response in the mouse model [175]. 

Man is an approved dietary supplement in the treatment of MPI-CDG in both Europe and the USA.

##### PMM2-CDG

PMM2-CDG (MIM: 212065) results from mutations in the gene encoding phosphomannomutase 2 (PMM2, EC 5.4.2.8), an enzyme that converts Man-6-P to mannose-1-phosphate (Man-l-P) in the cytoplasm. Phosphomannomutase 1 (PMM1) is a paralogous enzyme whose role, if any, in PMM2-CDG pathology has not been elucidated yet [191,192,193]. Prior to the identification of the underlying molecular cause of PMM2-CDG, Panneerselvam and Freeze had shown that the hypoglycosylation phenotype in PMM2-CDG patient fibroblasts could be resolved by the addition of Man to the culture medium [46]. This has been replicated by other in vitro [47,52,194] and in vivo models [77] (Figure 1).

In PMM2-CDG patients, Man appeared to be well-absorbed, increasing the blood levels of this sugar without any associated renal and/or hepatic toxicity [195,196]. Nonetheless no clinical or biochemical improvement was observed in these patients [196,197,198]. Despite this absence of glycosylation correction, some parents have reported improved psychomotor function in their children [199]. 

It has been postulated that PMM2-CDG cells have a reduced guanosine diphosphate (GDP)-Man pool, and that exogenous Man restores that depletion, hence, correcting the underglycosylation defect of these cells [52]. The direct correlation between phenotypic severity and protein truncation, manifested by lower mannose peak concentrations, suggests that the effectiveness of exogenous Man supplementation is influenced by PMM2 residual activity [59,198,199]. However, the presence of alternative transport systems cannot be excluded. Interestingly, it has been reported that metformin can induce and increase Man uptake by *PMM2*-deficient cells, by the activation of a Man-selective transport system, which corrects *N*-glycosylation in these cells [200].

To stimulate and solve uptake issues, maximizing retention and incorporation of Man, some alternative methods have been developed, including the synthesis of membrane permeable, hydrophobic Man-1-P-based prodrugs [201,202,203]. This could potentially improve stability, dissemination, assimilation, and therapeutic action of Man derivatives. In fact, hydrophobic Man-1-P compounds have corrected glycosylation in vitro [202].

The most recent strategy for Man supplementation is currently under development by Glycomine, which is developing a Man-1-P pharmacological formulation using liposomes as the delivery system [204].

#### 4.1.2. Defects in Monosacharide/Nucleotide Synthesis

##### CAD-CDG

**Figure 2:**
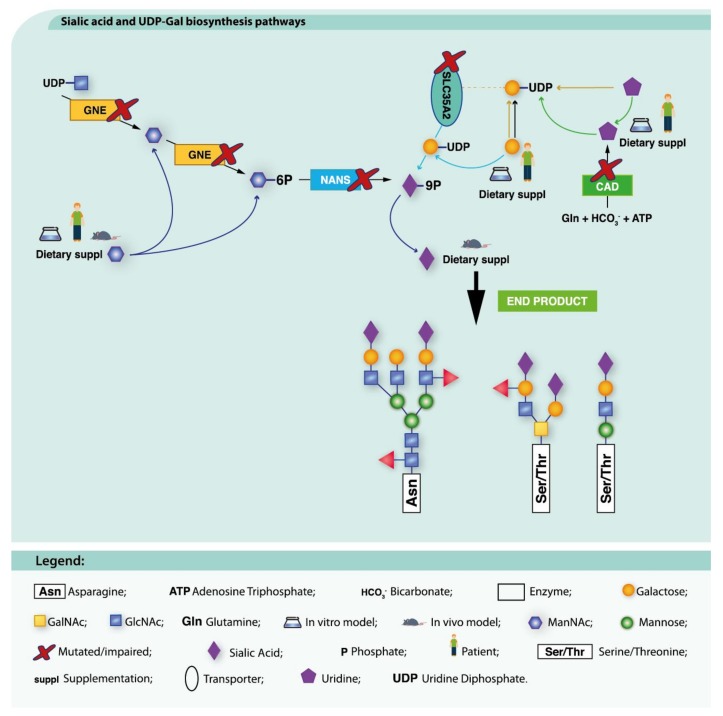
Dietary supplementation approaches under investigation for CAD-CDG, GNE-CDG, NANS-CDG and SLC35A2-CDG. Uridine supplementation has been tried for CAD-CDG (green arrow). Combined administration of uridine and Gal aims to increase Gal-UPD levels (yellow, green, black and light blue arrows). This allows the bypass of SLC35A2 transporter defect. ManNAc supplementation (dark blue arrow) has been used for GNE-CDG and NANS-CDG. These therapeutic approaches aim to recover the metabolic pathways (black arrows) and ultimately, the normal glycosylation profile (bold black arrow).

Uridine monophosphate synthase (UMPS)-deficiency (hereditary orotic aciduria, MIM: 258900) is a rare inborn error of metabolism (IEM) resulting in abnormal pyrimidine synthesis and uridine supplementation has shown positive clinical results in these patients [205]. *CAD* encodes a multifunctional enzyme complex (comprising amidophosphoribosyltransferase, EC 2.4.2.14; carbamoyl-phosphate synthase, EC 6.3.5.5; aspartate carbamoyltransferase, EC 2.1.3.2 and dihydroorotase EC 3.5.2.3) that catalyzes the first steps of de novo pyrimidine biosynthesis (Figure 2). Mutations in this gene have recently been associated with CDG (MIM: 616457) [69,100]. 

Based on this molecular knowledge and on previous findings obtained from in vitro CAD models, in which the decreased pools of nucleotide sugars (UDP-GlcNAc, UDP-GalNAc, UDP-Glc, UDP-Gal) and other metabolites that serve as glycosylation donors were normalized by uridine supplementation to the culture medium [100], Koch et al. treated two severely affected CAD-CDG patients with 100 mg/kg per day oral uridine in a daily regimen of 4 doses. This led to a dramatic clinical improvement, including seizure cessation, cognitive and motor development, increased alertness and communication as well as normalization of biochemical parameters [69].

##### GNE-CDG

GNE-CDG (MIM: 605820) is caused by defects in UDP-*N*-acetylglucosamine 2-epimerase (UDP-GlcNAc 2-epimerase, EC 5.1.3.14)/*N*-acetylmannosamine kinase (ManNAc kinase, EC 2.7.1.60), a bifunctional and rate-limiting enzyme of SA biosynthesis. Evidence that supplementation of SA precursors, such as the aminosugars ManNAc or d-mannosamine (ManN), can bypass GNE defects has been obtained in a set of different disease models (Figure 2) [19,74,78,81,109,110,206,207]. ManNAc treatment restored glycosphingolipid (GSL) levels in patient cells [66] and up-regulated *Gne* and protein expression, therefore suggesting that this aminosugar may act as a protein stabilizer [80,81]. As many GNE-CDG patients are likely to be diagnosed only in adulthood, the effects of oral ManNAc, SA or ManN therapy in adult mutant mice were tested, demonstrating that all of these compounds rescued kidney and muscle hyposialylation [78]. However, only ManNAc improved proteinuria [78,117]. 

Due to rapid degradation, clearance and, hence, low incorporation of the aminosugars, Ac_4_ManNAc and SA were also evaluated in vitro and in vivo with Ac_4_ManNAc showing the best results [82,83,207]. 6′-sialyllactose has also been administered to GNE-CDG mice. This compound is slowly metabolized which increases its blood half-life and cellular incorporation. It significantly ameliorated muscle functioning, strength and sialylation [82,84]. These preclinical results have been the basis for several clinical trials (see Table 3).

##### NANS-CDG

NANS-CDG (MIM: 610442) results from defects in the CMP-*N*-acetylneuraminic acid synthetase (EC 2.7.7.43), an enzyme involved in the SA biosynthetic pathway (Figure 2). In a zebrafish model, addition of SA to the water following morpholino injection led to a partial recovery of skeletal anomalies, but only when added 24 h post-fertilization [122]. These preliminary results should encourage the setting up of clinical trials, provided that further molecular, pharmacokinetics and pharmacodynamics data are gathered. However, they also raise the possibility of (in)effectiveness of post-natal therapy. A first-in-human trial using a slow release form of SA supplementation in NANS-CDG patients has been submitted for approval; however based on the negative results of SA therapy in GNE-CDG (Table 3), the slow release form of SA is currently not available for therapeutic trials.

##### PGM1-CDG

PGM1-CDG (MIM: 614921) is a disease affecting phosphoglucomutase 1 (PGM1, EC 5.4.2.2). PGM1 catalyzes the conversion of glucose-1-phosphate (Glc-1-P) to glucose-6-phosphate (Glc-6-P) and is a key enzyme for glycogen, glucose and galactose metabolism and glycosylation. Gal supplementation has restored *N*-glycosylation in vitro [65,208] and oral Gal consumption is safe and associated with significant improvement of *N*-glycosylation and clinical parameters (liver, coagulation and hormonal function) in PGM1-CDG patients [65,208,209] (Figure 2). 

**Figure 3:**
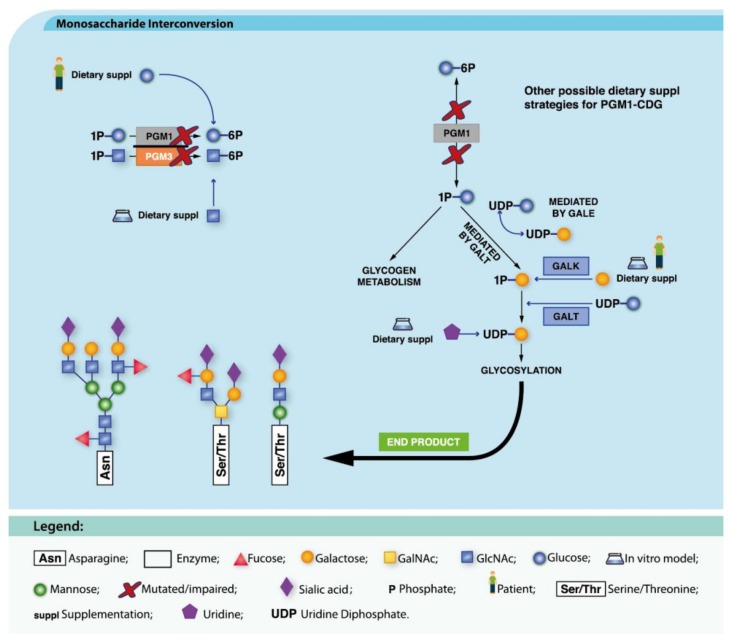
Dietary supplementation approaches under investigation for PGM1-CDG and PGM3-CDG. GlcNAc supplementation has been tried in vitro for PGM3-CDG (blue arrow) while for PGM1-CDG, Glc, uridine and Gal (blue arrows) have been tried. These therapeutic approaches aim to recover the metabolic pathways (black arrows) and ultimately, the normal glycosylation profile (bold black arrow).

Combined Gal and uridine (as a source of UDP-Gal, Figure 3) therapy has been attempted, with promising results in vitro [65]. Nevertheless, other symptoms, such as dilated cardiomyopathy (DC), are not corrected by Gal or its combination with uridine [209,210]. 

Exercise intolerance with episodes of rhabdomyolysis and hypoglycemia has also been associated with PGM1 deficiency and Gal and glucose administration has improved exercise tolerance [211,212].

##### PGM3-CDG

PGM3-CDG is due to defects in the phosphoglucomutase 3 (PGM3, EC 5.4.2.2) enzyme that catalyzes the interconversion of *N*-acetylgalactosamine-1-phosphate (GlcNAc-1-P) and *N*-acetylgalactosamine-6-phosphate (GlcNAc-6-P) [213]. *PGM3* defects cause a form of immunodeficiency (MIM: 615816), partially responsive to antimicrobial prophylaxis, but not to immunoglobulin replacement. *PGM3* mutations disrupt the synthesis of UDP-GlcNAc (an *N*-glycan, *O*-glycan, proteoglycan and GPI-anchored protein building block). Concordantly, GlcNAc supplementation to patients’ cells restored depleted UDP-GlcNAc, thus creating a therapeutic avenue for PGM3-CDG patients (Figure 3) [67]. Unfortunately, the therapeutic trial was unsuccessful, and the clinical trial for this approach was recently closed.

### 4.2. Defects Located in the Endoplasmic Reticulum (ER)

#### 4.2.1. Defects in Protein *N*-Glycosylation

##### ALG1-CDG

ALG1-CDG (MIM: 608540) is caused by defective LLOs mannosylation. The *ALG1* gene encodes the chitobiosyldiphosphodolichol β-mannosyltransferase (EC 2.4.1.142) which synthetizes β-1,4-d-mannosylchitobiosyldiphosphodolichol. After a 16h incubation with 500 μmol/L of Man, ALG1-CDG patient fibroblasts displayed increased levels of high-mannose (Man) and sialylated *N*-glycans, as well as decreased levels of the *N*-tetrasaccharide, which was put forward as a disease biomarker [178]. This encouraging data not only reveals the existence of a promising, therapy-sensitive biomarker, but also points out sugar supplementation as a possible therapeutic approach for these patients.

##### ALG13-CDG

ALG13-CDG (MIM: 300884) is due to the impairment of *N*-acetylglucosaminyldiphosphodolichol *N*-acetylglucosaminyltransferase (EC 2.4.1.141), encoded by *ALG13* which leads to *N*-glycosylation deficiency. In fibroblasts from a patient with a hemizygous missense mutation (p.E463G), d-galactose (Gal) addition to the culture media increased low ICAM-1 levels to almost normal values [190]. This points to ICAM-1 as a potential disease and therapy informative biomarker, and Gal supplementation as a putative treatment for ALG13-CDG patients.

##### MAGT1-CDG

The role of magnesium transporter 1 (MAGT1) as a Mg^2+^ transporter has been long established [90], but the importance of this protein in *N*-glycosylation has only been recently unraveled [214]. *MAGT1* mutations have been associated with a X-linked immunodeficiency with Mg^2+^ defect, Epstein-Barr virus (EBV) infection and neoplasia (XMEN syndrome, MIM:300853) [63,215]. In vitro and in vivo Mg^2+^ supplementation has shown encouraging results [63,215] and a patient has been reported to be on oral Mg^2+^ therapy (Figure 3). Unfortunately, no patient follow-up has yet been reported.

#### 4.2.2. Defects in Lipid Glycosylation and GPI Synthesis 

##### PIGA-CDG

*PIGA* encodes the subunit A of the phosphatidylinositol *N*-acetylglucosaminyltransferase enzyme (EC 2.4.1.198). Defects in this catalytic subunit result in the multiple congenital anomalies-hypotonia-seizures syndrome-2 (MCAHS2; MIM: 300868) characterized by brain and other organ affectations [216].

Two brothers with drug-resistant seizures showed great improvement upon initiation of a ketogenic diet, including seizure control and improved development [216]. Ketogenic diet has high fat and low carbohydrate content, therefore making ketone bodies the brain’s main source of energy [217]. Ketone bodies stimulate γ-amino butyric acid (GABA) production and reception, initiating an antiepileptic effect [158].

Ketogenic diets are usually rich in omega-3 and omega-6 polyunsaturated fatty acids (PUFAs) [218]. PUFAs have recently been described to have modulatory effects on voltage-gated ion channels (transmembrane proteins essential for heart and brain function), and thus, a potential anti-epileptic effect [218]. One should keep in mind that a ketogenic diet may aggravate/potentiate hypoglycemia in these patients [15].

##### PIGM-CDG

**Figure 4:**
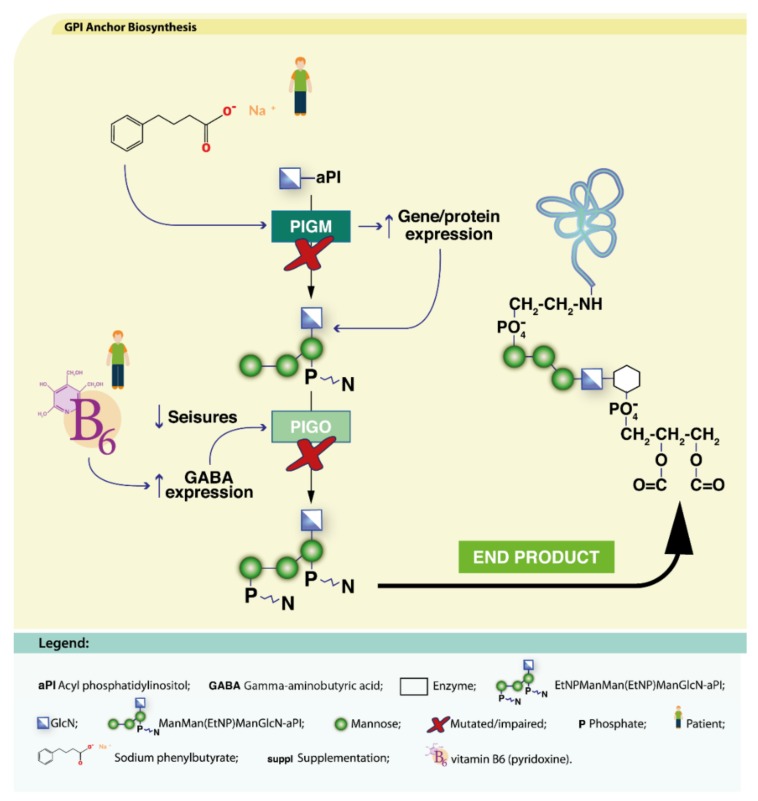
Dietary supplementation approaches under investigation for PIGM-CDG and PIGO-CDG. Vitamin B6 due to its ability to increase GABA expression has been tried for PIGO-CDG (blue arrows). Sodium butirate, which can increase *PIGM* expression, has been tried for PIGM-CDG (blue arrow). These therapeutic approaches aim to recover the metabolic pathways (black arrows) and ultimately, the final end product (bold black arrow).

PIGM-CDG (MIM: 610293) results from mutations in the promoter region of the *PIGM* gene. *PIGM* encodes the catalytic subunit of the GPI α-1,4-mannosyltransferase I enzyme and defects in this protein results in deficient addition of the first Man in the GPI core biosynthesis [219]. This disrupts binding of the Sp1 transcription factor, impairs histone acetylation, gene transcription and expression. Almeida et al., described that sodium butyrate induced acetylation, and increased *PIGM* transcription, both in vitro and in a patient (Figure 4). The patient became seizure-free, and reacquired previously lost capabilities [57]. These data suggest that histone deacetylase (HDAC) inhibitors may be an interesting therapeutic approach for this CDG [219].

##### PIGO-CDG

PIGO-CDG (MIM: 614749) is a defect in the *PIGO* gene that encodes the GPI glycan biosynthesis class O (also known as GPI ethanolamine phosphate transferase 3) protein which is involved in GPI-anchor biosynthesis [35]. Defects in PIGO cause hyperphosphatasia and intellectual disability. Similarly to what happens in PIGA-CDG, PIGO-CDG patients suffer from intractable seizures. Oral administration of 400 mg (20 mg/kg) of vitamin B6 (pyridoxine) has rendered a PIGO-CDG patient seizure-free [220]. The therapeutic effect of vitamin B6 intake is likely related to stimulation of brain GABA synthesis, which may be impaired due to lack of pyridoxine [35] (Figure 4).

### 4.3. Defects Located in the Golgi Apparatus

#### 4.3.1. Defects in Nucleotide-Sugar Transporters

##### SLC35A1-CDG

SLC35A1-CDG (MIM: 603585) is caused by defects in the solute carrier family 35 member A1 protein which transports CMP-SA to the Golgi complex. Addition of SA sources, such as SA itself, ManNAc or the glycoprotein fetuin, to the culture media failed to rescue the sialylation deficiency in patient-derived cells [71].

##### SLC35A2-CDG

SLC35A2-CDG (MIM: 300896) arises from defective UDP-Gal transportation to the Golgi apparatus by the UDP-Gal translocator (also known as solute carrier family 35 member A2). Gal supplementation (1 g/kg/day) in a patient normalized the transferrin glycosylation profile after 6 months (Figure 2) [33]. Remarkably, in a previous study using patient-derived fibroblasts, Gal addition to the culture media did not achieve any results [221]. This might be partly explained by different experimental conditions and/or by distinct genetic backgrounds.

##### SLC35C1-CDG

SLC35C1-CDG (MIM: 266265) is caused by defects in the *SLC35C1* which encodes a GDP-fucose transmembrane transporter. This leads to deficient import of GDP-fucose into the Golgi apparatus. Fucose addition to the culture medium corrects fucosylation as well as other biochemical and functional parameters in cells [23,27,50,51,53], and in animal models [132,136]. Fucose supplementation produced biochemical and clinical improvement, including normalization of neutrophil counts, re-expression of E- and P-selectin ligands, infection recurrence cessation [23,53,54,222], as well as improved psychomotor development, although to a lesser extent [23,53,54]. Divergence in therapy efficacy and effect has also been reported. SLC35C1-CDG patients have the Bombay blood types, as they lack the α1,2-fucosylated H-antigen. Fucose therapy did not result in the expression of the H antigen in one patient [53], while in another, expression of the H antigen was induced by therapy. Although no autohemolysis was seen in this patient, the potential for autoimmune reactions triggered by fucose therapy in SLC35C1-CDG has to be considered [23]. Moreover, no therapeutic benefit has been reported in some patients [55,223]. These response differences could be due to differences in mutational background [22], residual enzymatic function, overall clinical severity [23] or cellular mislocalization [27]. Further molecular and clinical data is warranted to further clarify the therapeutic potential of fucose administration to SLC35C1-CDG patients.

#### 4.3.2. Other Defects

##### TMEM165-CDG

**Figure 5:**
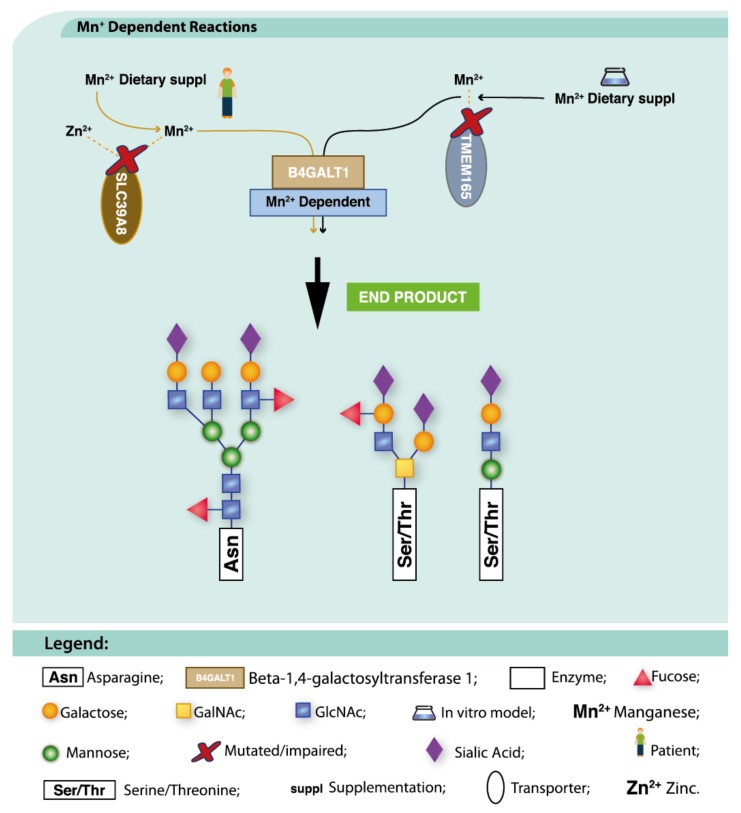
Dietary supplementation approaches under investigation for TMEM165-CDG and SLC39A8-CDG. Impaired transport of metal ions (dotted yellow lines), can be circumvented by Mn^2+^ supplementation (yellow and black lines). These therapeutic approaches aim to recover the metabolic pathways (black arrows) and ultimately, the final end product (bold black arrow).

TMEM165-CDG (MIM: 614727) is a defect in a manganese (Mn^2+^) transporter of the Golgi apparatus (Figure 5). Mn^2+^ supplementation has suppressed the glycosylation defect in vitro [142] and daily intake of Gal (1 g/kg/day) led to improved biochemical parameters and *N*-glycosylation in two patients harbouring the same homozygous mutation and with normal Mn^2+^ levels, [70]. However, two different mutations R126H and E108G displayed altered Mn^2+^ sensitivity. This finding highlights the potential impact of distinct genetic alterations on therapy responses [36].

### 4.4. Defects Located in the ER-Golgi Intermediate Compartment( ERGIC)

#### 4.4.1. Defects in Multiple and Other Glycosylation Pathways

##### CCDC115-CDG

CCDC115-CDG (MIM: 616828) is caused by defects on the coiled-coil domain containing protein 115, one of the subunits of the Vacuolar H^+^ ATPase (V-ATPase), the key proton pump for endo-lysosomal acidification. Disruption of V-ATPase function has been related to descreased intracellular iron levels which desrupt iron(II) prolyl hydroxylase (PHD) enzymes, activate hypoxia inducible transcription factors (HIFs) and impair transferrin uptake [106]. It has also been related to defective homeostasis of the Golgi apparatus [106,224]. CCDC115-CDG patients display a type 2 transferrin IEF, psychomotor disability, hypercholesterolemia, hypotonia and predominant liver involvement [3,224]. Supplementation of a *CCDC115* knockout cellular model with iron (Fe(III)) citrate restored PHD enzymatic activity and HIF1α turnover [106]. Replication of these experiements using patient-derived material would elucidate if iron supplementation could represent a therapeutic approach for these patients.

##### TMEM199-CDG

TMEM199-CDG (MIM: 616829) like CCDC115-CDG is caused by defects on one of the subunits of V-ATPase and indications suggest that TMEM199 and CCDC115 form a complex [106]. TMEM199-CDG patients present with mild liver dysfunction [225] and as for CCDC115-CDG, iron supplementation should also be investigated on patient-derived material to assess potential therapeutic value.

### 4.5. Defects Located at the Plasma Membrane

#### 4.5.1. Defects in Multiple and Other Glycosylation Pathways

##### SLC39A8-CDG

SLC39A8-CDG (MIM: 616721) is a disorder in Mn^2+^, zinc (Zn^2+^) and cadmium (Cd^2+^) transport, with a secondary impact on glycosylation due to the dependence of β-1,4-galactosyltransferases on Mn^2+^ (Figure 5). A multi-agent, phased therapeutic approach composed of Gal, uridine (to ensure sufficient UDP necessary for UDP-Gal synthesis), and eventually with the addition Mn^2+^ (due to undetectable levels of this element in the blood of the patient) has resulted in improved glycosylation after 2 weeks [226]. Combined treatment with both Gal and uridine has also shown substantial normalization of the glycosylation profile of a SLC39A8-CDG patient with a severe phenotype [227]. A follow-up of two patients on Mn^2+^ monotherapy for over 12 months showed significantly improved biochemical and clinical manifestations [228]. Although Gal therapy corrects the glycosylation defect, it is incapable of overcoming the impact of the lack of Mn^2+^ in Mn^2+^-dependent enzymes and processes [228].

### 4.6. Defects Located at the Sarcolemma Membrane

#### Defects in *O*-Mannosylglycan Synthesis

##### ISPD-CDG

**Figure 6:**
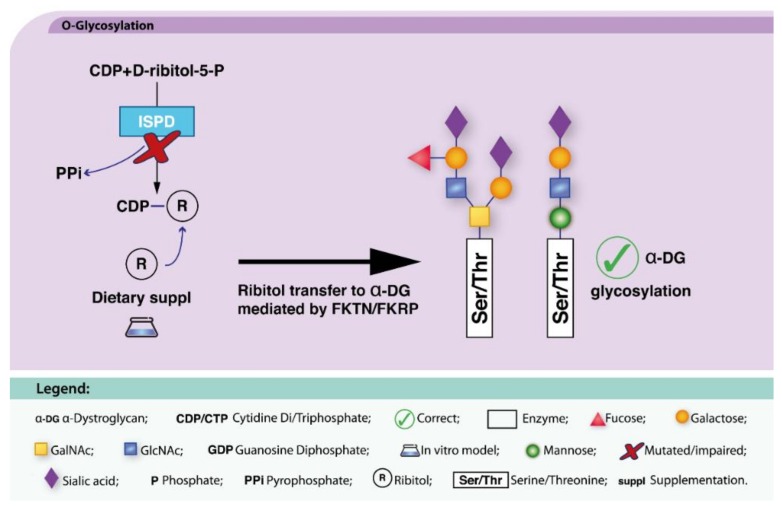
Dietary supplementation approaches under investigation for ISPD-CDG. ISPD synthetizes CDP-ribitol from CDP and D-ribitol-5-P, with the release of PPi (black and blue arrows). Ribitol supplementation (blue arrow) aims to circumvent ribitol shortage and ultimately the recovery of α-DG glycosylation (bold back arrow).

ISPD-CDG (MIM: 614643, 616052) is caused by mutations in the *ISPD* gene. *ISPD* encodes the 2-C-methyl-d-erythritol 4-phosphate cytidylyltransferase that synthesizes CDP-ribitol [32,68]. Defects in *ISPD* lead to impaired α-dystroglycan (α-DG) *O*-mannosylation. In two elegant in vitro studies, addition of ribitol or ribitol-metabolites (CDP-Rbo) to the culture medium significantly increased α-DG glycosylation, and restored laminin binding ability (Figure 6). Thus, ISPD-CDG patient-derived cells reacquired the capability to be glycosylated as well as their normal morphology [68,150], highlighting ribitol as a candidate therapeutic compound.

## 5. Other Therapeutic Strategies 

Although supplementation either with sugars or other molecules has been successful in some CDG, the majority of these disorders remain without treatment and hence, other strategies are being pursued. 

### 5.1. Pharmacological Chaperones

Proteins exist in the intracellular medium in equilibrium between folded and unfolded states. Point mutations can shift this equilibrium towards the unfolded state inducing protein destabilization and aggregation [229]. Pharmacological chaperones (PCs) are small molecules that bind specifically to a protein and shift the equilibrium towards the folded state [230]. PCs can bind to the active site acting as competitor inhibitors or to allosteric sites of the protein and have no inhibitory effect [231]. 

PMM2-CDG has been classified as a misfolding disorder since the majority of mutations described are of the missense type and most mutants retain some residual enzymatic activity. In silico docking has demonstrated that it is possible to find ligands that stabilize PMM2 [232]. Thus, PCs present as a promising therapy [232,233,234,235,236]. Recently, a high-throughput screening of a commercially available library of 10,000 low molecular-weight compounds led to the identification of eight compounds that specifically stabilized the PMM2 protein. Further studies using oligomerization mutants, a cell-based disease model over-expressing the p.Asp65Tyr, p.Pro113Leu, p.Arg162Trp, and p.Thr237Met PMM2 mutants and in silico analysis narrowed the number to one compound, 1-(3-chlorophenyl)-3-3-bis(pyridine-2-yl)urea (compound VIII), that presented a viable chemical structure for further optimization [231]. These results represent the first proof-of-concept that PCs could be used as treatment for PMM2-CDG. 

Another therapeutic strategy proposed for PMM2-CDG is the inhibition of MPI which is based on fact that the majority of Man-6-P is catabolized by MPI reducing the amounts of this precursor available for glycosylation [237]. A potent MPI inhibitor, MLS0315771, derived from the benzoisothiazolone series was shown to direct Man-6-P toward glycosylation in several different cell lines, including PMM2-CDG patient-derived fibroblasts, improving *N*-glycosylation [59,237]. Despite this positive outcome, the effect of MLS0315771 was dependent on the mutation type and the levels of residual PMM2 mutant activity, since five of the nine patient-derived cell lines did not present any improvement. This might be due to the presence of the p.Arg141His mutant, responsible for a complete lack of enzymatic activity. However, improvement was observed in one cell line bearing the p.Arg141His and p.Cys241Ser mutants, although residual activity was lower compared with other patient-derived cell lines which did not show improvement [59].

The effect of MLS0315771 was also tested in zebrafish embryos. An inhibitory effect of MPI was observed, although with toxic effects for concentrations above 2 µM. Thus, further compound optimization is necessary in order to maintain MPI inhibition while reducing toxicity [59]. 

### 5.2. Antisense Therapy

Although less frequent, the disruption of correct splicing by point mutations is estimated to represent about 15% of all the mutations described in the Human Gene Mutation Database (HGMD^®^) [238]. The disruption of conserved intron-exon junction sequences or other intronic regions, can lead to activation of cryptic splice-sites and inclusion of intronic sequences called pseudoexons [238,239]. Antisense therapy using morpholino oligonucleotides (AMO) has been tested in patient-derived fibroblasts for PMM2-CDG and TMEM165-CDG [238,239]. 

Several mutations affecting normal splicing of *PMM2* have been described [239,240,241]. Vega et al. [239] described the use of AMOs designed towards the donor and acceptor cryptic splice sites of a pseudoexon activated by c.640-15479C>T mutation to treat patient-derived fibroblasts. A normal splicing profile was obtained 24 h post-transfection in a sequence and dose-dependent manner and both protein amount and enzymatic activity were restored by 30 to 45% [239].

A deep intronic change, c.792+182G>A leading to a pseudoexon insertion affecting the TMEM165 protein was described by Yuste-Checa et al. [238]. A specific AMO sequence targeting the intronic 5′ cryptic splice site was used, allowing the recovery of normal splicing profile and protein levels. AMO treatment also resulted in increased protein expression in the Golgi apparatus [238]. 

### 5.3. Gene Therapy

Since CDG are a group of monogenic disorders, they are potential candidates for gene therapy. In fact, in different disease model and patient cells, introduction of the normal copy of a faulty gene rescued the disease phenotype [34]. Gene therapy consists in the successful transfer and activation of a fully functional copy of an aberrant gene [242] in a patient cell system. In order to achieve this goal, a safe vehicle that protects the gene copy must be used. In comparison with adenoviral and retroviral vectors, adeno-associated virus (AAV) vectors represent a safer approach [242]. Gene therapy using the adeno-associated virus type 8 (AAV8) as vehicles for human *GNE*, was studied as a possible therapeutic strategy in GNE-CDG. Human and murine GNE myopathy muscle cells were transduced with the virus and they successfully expressed the transgene [61]. These results were also replicated in an animal model [243] with increased GNE expression in skeletal muscle, liver, kidney, heart and spleen 10 weeks after the injection. Mice were also injected at 47 weeks of age showing significant improvement in survival, motor and contractile performance and muscle size [243]. C57B16 wild-type mice between five and six weeks old were injected with two doses of the transgene which maintained a sustained expression for the six months follow-up period, without toxic effects [61]. 

Concerns regarding the possible adverse effects of GNE over-expression, led to another approach using an AAV-based *trans*-splicing (TS) mechanism to overcome the *GNE* M712T mutation [244]. TS can occur in mammals and is mediated by the spliceosome allowing the slicing of two different pre-mRNA molecules. The advantage of this system is the ability of correcting transcript defects by altering normal splicing process, while maintaining the original endogenous gene regulation [244]. The AAV-TS system was tested in human GNE myopathy muscle cells carrying the M712T mutation and was able to generate wild-type *GNE* transcripts, although with low efficiency [244]. 

AAV vector-targeted immune responses remain a major limitation of this gene-delivery tool in clinical practice. Different approaches such as liposomes or exosomes are being explored as safer delivery systems [245]. 

Phadke et al. [246], described the use of a GNE expressing vector complexed with a cationic liposome (GNE-lipoplex). The same vector had already successfully increased SA expression by transient transfection of GNE-deficient CHO-Lec3 cells lines [28].

BALB/c mice were used to assess toxicity and GNE-lipoplex was administered intramuscularly (IM) or intravenous (IV) in a single dose of 10, 40 or 100 µg. No adverse effects were observed with the intermediate dose of GNE-lipoplex administered either IM or IV, with detection of human GNE mRNA in mice tissues [246]. GNE-lipoplex was also tested in a severely affected patient with acceptable safety and promising results [247,248]. 

### 5.4. Transplantation Options

Symptom severity, disease progression and the lack of better suited therapeutic options have prompted organ transplantation as viable treatment option for IMDs [249,250,251]. 

#### 5.4.1. Liver Transplantation

Liver involvement is present in several CDG (reviewed in Marques-da-Silva et al. [3]). Janssen et al. report the first instance of liver transplantation in a MPI-CDG patient [171]. The patient presented hepatomegaly and developed congenital hepatic fibrosis and portal hypertension with hepatic vein thrombosis. Oral mannose supplementation (1 g/kg/day) was initiated at the age of 15 years, with some improvement, but due to therapy-resistance and progressive liver failure the patient was accepted for transplantation followed by major improvement [171]. 

Liver transplantation was also performed in two CCDC115-CDG patients [224]. The patients were siblings and presented hepatosplenomegaly, elevated serum transaminases and alkaline phosphatase. Both received liver transplants but unfortunately, the brother rejected the transplant twice and died. The sister is doing well after the procedure with normalization of serum aminotransferase levels and transferrin glycosylation profile [224]. 

Currently, liver transplant is an approved therapy in Europe for MPI-CDG, CCDC115-CDG and ATP6VAP1-CDG patients, and since the initially reported patients, additional () patients have been transplanted.

#### 5.4.2. Heart Transplantation

The heart can also be affected in CDG patients (reviewed in Marques-da-Silva et al. [9]). Kapusta et al. reported cardiac pathology in nine patients diagnosed with DOLK-CDG (MIM: 610768), caused by dolichol kinase deficiency [252]. Cardiac manifestations varied from mild dilation to heart failure with death. Two of the patients died with acute symptoms of heart failure before CDG diagnosis, four patients diagnosed with mild DC were treated with supportive heart failure therapy (ACE inhibitors, β blockers and diuretics), while three patients also with mild DC received heart transplants due to rapid deterioration. Despite a successful procedure, one of the patients died unexpectedly while in the other two, the symptoms stabilized [252]. Based on the successful transplantation in several mild patients [253], heart transplantation is an approved therapy for the pure cardiac form of DOLK-CDG.

Heart transplantation was also reported as an option for PGM1-CDG patients by Tegtmeyer et al. [65]. From a cohort of nineteen patients, six presented DC, cardiac arrest or both. Three of them were listed for heart transplantation [65]. 

#### 5.4.3. Cell Transplantation 

Hematopoietic stem cell transplantation from cord blood and bone marrow has been described as a lifesaving treatment for two PGM3-CDG affected children [42]. Both patients presented severe immunodeficiency which was successfully cured by the transplants [42]. Stem cell transplantation is currently an approved therapy for these patients in the USA.

Additionally, stem cell transplantation was planned for a MAGT1-CDG patient. The outcome of the procedure has not (yet) been reported [215].

## 6. Observational and Interventional Clinical Trials

The number of curative treatments for CDG and IMDs in general remains low [254]. Clinical trials, aimed to discover or verify the potential effects of a determined treatment, are crucial steps towards drug therapy approval, but several pitfalls exist in developing clinical trials for IMDs, especially related to the reduced number of subjects and the proposal of control groups for randomized controlled trials [254]. 

### 6.1. Natural History Studies

To assess the effects of a possible curative treatment it is essential to establish solid and quantifiable end-points based on patients’ natural history. In CDG, this task can be especially difficult due to the broad range of different clinical manifestations observed. 

In order to document the natural history of CDG patients accounting for the multisystem aspects of disease, researchers have developed and validated several rating scales designed to monitor disease progression.

The Nijmegen Paediatric CDG Rating Scale (NPCRS) is based on clinical symptoms and is composed of three sections of questions related to the disabilities suffered by CDG-affected children up to the age of 18 years. [255,256]. 

Despite evaluation of CNS involvement by the NPCRS, according to Serrano et al. [257], the cerebellar symptoms observed in PMM2-CDG patients are not specifically addressed. To address this, the International Cooperative Ataxia Rating Scale (ICARS), developed to assess cerebellar ataxia, was validated in a cohort of 13 patients [257] and also in a follow-up study of 20 patients [258]. Although ICARS has only been applied to PMM2-CDG, it could be transposable to all CDG with cerebellar involvement. MRI measurements [midsagittal vermis relative diameter (MVRD) and volume] for disease progression assessment have also been used [259].

A rating scale specific for PGM1-CDG was designed also based on clinical symptoms to grade disease severity and guide physicians in the diagnostics and prognosis counseling [260]. The Tulane PGM1-CDG Rating Scale (TPCRS) is structured similarly to the NPCRS and allowed the classification of a cohort of 27 PGM1-CDG patients [260]. 

In order to optimize care for GNE-CDG patients and also improve the design of clinical studies, the GNE Myopathy Functional Activity Scale (GNEM-FAS) was created [261]. This 25-item questionnaire was successfully applied to 47 subjects undergoing a Phase 2 study with extended release SA [261]. Another study of natural history in a cohort of 24 Japanese GNE-CDG patients was reported by Mori-Yoshimura et al. [262]. In order to broaden the investigation of clinical conditions and establish a long-term follow-up, a Japanese national GNE myopathy patient registry (Registration of Muscular Dystrophy; REMUDY) was created [262,263]. 

Several observational clinical trials have been registered in order to collect patient information for further studies (Table 4). 

### 6.2. Interventional Clinical Trials

Several trials regarding dietary supplementation therapy for CDG are in clinical phase and are summarized in Table 4.

#### 6.2.1. GNE-CDG

The effect of ManNAc in GNE-CDG patients was studied in a phase 1, randomized, placebo-controlled, double-blind study (clinicaltrials.gov NCT01634750) [264]. Based on the results of this clinical trial a regimen of 6 g administered twice a day was selected for future clinical trials [264]. The effects of 3 g and 6 g ManNAc administered twice a day for a time frame of 120 days is under study in an open label, phase 2 study (clinicaltrials.gov NCT02346461). 

The administration of Ace-ER tablets with a proper formulation has also been studied in phase 1 and 2 clinical trials [265], (clinicaltrials.gov NCT01359319, NCT01517880). These showed that this formulation increased serum free SA levels, with significant improvement of muscle strength in the upper extremities and no serious adverse effects reported. To further confirm the results obtained, an international randomized, double-blind, placebo-controlled phase 3 study is being conducted (clinicaltrials.gov NCT02736188). The results of this trial have not been reported yet. 

The possible therapeutic effects of immune globulin were also studied in a cohort of 4 patients (clinicaltrials.gov NCT00195637) [266]. Patients presented qualitative improvement for approximately three weeks, after which a decline in function was described [266]. 

#### 6.2.2. PGM1-CDG

The effect of Gal supplementation in PGM1-CDG patients was evaluated in a cohort of nine individuals in a pilot study [208] (clinicaltrials.gov NCT02955264). Treatment was well tolerated and no adverse events, aside from gastroenteritis in one patient, were registered. Liver function and coagulation parameters improved. Serum transferrin glycosylation improved in 8 patients. One patient continued Gal therapy (1 g/kg/day) for an additional period of 12 months during which all the parameters that improved and normalized during the trial remained stable [208]. Despite the positive results reported it is important to highlight that the study sample was small and laboratory baseline values were inconsistent in several function tests [208]. Gal treatment is now an approved dietary supplementation for the therapy of PGM1-CDG in Europe and the USA.

Patients with other CDG have also been included in this currently ongoing clinical trial (NCT02955264).

## 7. Discussion

Due to the multisystem presentation of CDG a “one size fits all” therapeutic solution is not feasible. Knowledge on the specific pathophysiological aspects of each CDG is fundamental to achieve better diagnostics, disease management and therapeutic solutions. Patient-derived material has been extensively used for diagnosis and research, but it presents limitations and some findings cannot be replicated in patients (e.g., Man supplementation in PMM2-CDG) [46,47,52,194,198] while others may be misleading as demonstrated by the null effect of Gal supplementation in SLC35A2-CDG patient-derived fibroblasts [221]. The generation of patient-derived iPSCs [93] will allow the investigation of disease mechanisms influencing embryonic development. The possibility of organoid generation also opens a new research avenue in which organ specific disease mechanisms and therapeutic approaches can be investigated. The generation of animal models for CDG can be challenging since glycosylation is an essential process and its severe impairment may not be compatible with life causing embryonic and/or neonatal lethality [74,77,121,124]. Other factors should also be taken into consideration regarding animal models, such as human disease phenotype recapitulation and phenotypic variability, since different genetic backgrounds within the same species can cause different disease presentations [76,81,269]. 

In recent years, an increasing number of studies evaluating the potential of dietary supplementation in CDG have emerged encouraged by the success of Man therapy in MPI-CDG. Nutritional therapies present many advantages, like being inexpensive food supplements, easily available and afforded by the families. To date, dietary supplementation strategies have been experimented on 21 CDG types using different disease models with a wide-range of results. These therapies are also in distinct phases of development (Figure S1). Some have only been tested in vitro, while others have already been trialed in humans. The recognition of dietary supplementation strategies as approved therapies will reduce costs facilitating access to the patients.

Nutritional supplementation in CDG such as, CAD-CDG [69], GNE-CDG [264,265], PGM1-CDG [65] and SLC35C1-CDG [53,270] have shown positive results, which has fueled the interest in this field. Additionally, as more knowledge about the affected molecular mechanisms responsible for disease phenotypes is built, increasingly targeted and precise therapies—some based on dietary compounds—used either alone [228] or in combinatory schemes [65,200] are being developed. Despite this, much remains to be elucidated in terms of the uptake, bioavailability, half-life, excretion, and toxicity of dietary supplements. Other parameters, such as dosage, route of administration, effectiveness/safety of both pre-natal and post-natal therapy, as well as long term administration consequences also need optimization and clarification.

The development of personalized medicine based on the patients’ genotype rather than phenotype has led to other approaches. PCs have been developed for several disorders [229,230] and are based on the existence of a protein with some residual activity that can be recovered. Thus, severe point mutations affecting the catalytic site or that severely impair the oligomerization state of the protein are not amenable to this therapy. As for gene and antisense therapy, the pitfalls rely on a safe and efficient delivery mode that can reach both peripheral organs and cross the blood brain barrier (BBB), as many CDG present neurologic impairment. Regarding organ transplantation, issues related to donor/organ availability and procedure complications have to be taken in account. 

Solid preclinical investigation using both in vitro and in vivo models, stable and reliable disease biomarkers, good screening methods and extensive knowledge of disease natural history are the basis for clinical trials. Hence, the drug development process is a high cost and time consuming process. According to the European Medicines Agency (EMA), incentives for the development of Orphan drugs for IMDs have increased, in order to spike pharmaceutical companies’ interest. This has already resulted in an increase of the number of orphan drugs approved. Nevertheless, there is only about 10% chance that a new therapy will be successfully approved by government regulatory agencies [271] and even then there are no guaranties that these drugs will reach the target patients due to country specific reimbursement issues [272]. Drug repositioning which is based on the use of existing and approved drugs for other diseases in therapeutic areas different from those with marketing authorization has become a possible solution [271,273,274]. Acetazolamide, a carbonic anhydrase (CA) inhibitor, can be considered a successful case of drug repositioning, since it has been used in the prophylaxis and treatment of acute mountain sickness [275], hydroxychloroquine retinopathy [276], hyperphosphatemic familial tumoral calcinosis [277] and also in the treatment of disorders associated with ataxia [278]. Now it is being evaluated for cerebellar involvement in PMM2-CDG (Clinical trial identifier 2017-000810-44). 

However, to use this approach it is fundamental to be aware of different DNA sequence variants that cause or influence disease phenotype. Westphal et al. described a frequent mild mutation in *ALG6* that can influence disease severity of PMM2-CDG patients [279]. The same mutation was also identified in a case of CDG type I associated with low dehydrodolichol diphosphate synthase (DHDDS) [280]. This indicates that other unknown variants might be associated with disease mechanism. Genome-wide association, ChIP-seq and RNA-seq studies, allied to strong computational analysis can give valuable information regarding the expression of different genes in CDG patients-derived samples. 

Research advances during the last few years open new therapeutic avenues for CDG. In this review we have collected and selected information regarding several aspects involved in the development of therapies in order to facilitate research and increase information in this area. All the information collected regading disease models and the different therapeutic approaches is summarized in Table S1. By systematizing existing knowledge on and therapeutic solutions for CDG, we hope to intensify further research, and, at the same time promote patient-involvement in research, and ensure that CDG patients receive the best possible care.

## 8. Methods

For this review, a combination of specific keywords related to therapies, clinical trials, animal and cellular models, biomarkers and CDG was used to search the Medline database, using PubMed [281] as the search engine. After the data collection the possible duplicate registries were eliminated and a refinement of the results was performed based on the title and abstract of each publication. After this step, the selected articles were read and the ones matching the selection criteria were included. Inclusion/exclusion criteria were the following:

(a) Only English-written manuscripts were included;
(b) Articles reporting biomarkers, in vitro and/or in vivo models, compassionate use or clinical trials of therapies in CDG were included;
(c) Only articles reporting CDG with therapies under development (compassionate use, clinical research) or already approved were included;
(d) Reviews were excluded, although we have included some examples for contextualization purposes;

For information about clinical trials, both the European [268] and American [267] web pages were consulted. Information about mouse models was also retrieved from the Jackson Laboratories web page [86]. More information about the selection process can be seen in Figures S2 and S3.

## Figures and Tables

**Table 1:** Overview of congenital disorders of glycosylation (CDG) disease models.

Defect	CDG/Protein	Cell/Organism/Animal	Model	Major Findings/Phenotype	Reference
**Protein *N*-glycosylation**	ALG1-CDG/Chitobiosyldiphosphodolichol β-mannosyltransferase	*Saccharomyces cerevisiae*	*Alg1* mutants (K57-6C strain)	-Thermosensitive. -Increased levels of GDP-Man by Mannose-1-phosphate guanylyltransferase (MPG1) gene overexpression restores defects in mannosylation, in contrast to Man supplementation.	[24,34,41]
		*S. cerevisiae*	*Alg1* mutants (PRY56 strain)	-Thermosensitive.	[31]
	ALG6-CDG/α-1,3-glucosyltransferase	*S. cerevisiae*	*Alg6* mutants	-Unable to transfer glucose from dolichol phosphoglucose in the Lipid-linked oligosaccharides (LLO) synthesis leading to the accumulation of Man_9_GlcNAc. -Shorter LLO glucose chain causes growth defects. -*Alg6* mutants stopped growing completely.	[87,88]
		Chinese hamster (CHO) cell line	MI8-5 cells (*Alg6^−/−^*)	-Unable to synthetize glucosylated oligosaccharides.	[89]
	MAGT1-CDG/Magnesium transporter 1	Human embryonic kidney (HEK293) cell line	*Magt1* knockdown by siRNA	-Decreased Mg^2+^ uptake. -Combined *Magt1* and *TUSC3* overexpression raised cellular Mg^2+^ content.	[90]
		*S. cerevisiae*	*Alr1∆* strain	-Inability to proliferate in Mg^2+^ free medium is overcome by *magt1* complementation.	[90]
		*Dario renio* (zebrafish)	(a) Zygotic *Magt1* knockdown (b) Maternal and zygotic *Magt1* knockdown	(a) Embryonic lethality. Profound developmental abnormalities. (b) Inability to hatch. MgCl_2_ recovered lethality.	[90]
**Protein *N*-glycosylation**	MPI-CDG/Mannose-6-phosphate isomerase	Human colon adenocarcinoma (HT-29) cell line	*Mpi knockdown* by siRNA	-Inhibiting *Mpi* by 50–70% increases Man incorporation into proteins.	[59]
		*Mus musculus* (mouse)	*Mpi* (Mpi^−/−^) knockout	-Normal glycosylation profile. -High level of embryonic lethality that was exacerbated by Man supplementation due to Man-6-P accumulation and a decrease in adenosine triphosphate (ATP) levels.	[91]
		Mouse	Hypomorphic *Mpi*^Y255C/Y255C^	-Reduced in utero mortality which was increased by mannose supplementation to the pregnant dams. The surviving offspring presented severe ocular defects. -No phenotypic presentations.	[72]
		Zebrafish	*Mpi* mutant with 13% of enzymatic activity	-MPI-CDG biochemical and phenotypic presentations. -Addition of mannose to the fish water rescued *mpi* morphants phenotype but only if provided prior to 24 h post fecundation (hpf).	[92]
	PMM2-CDG/Phosphomannomutase 2	Human induced pluripotent stem cells (iPSC)	(a) Hypomorphic PMM2^422G>A/357C>A^-iPSC (b) Hypomorphic PMM2^422G>A/357C>A^-iPSC with additional knockdown by shRNA	-Reduced PMM activity, accumulation of shorter glycan structures and reduced mannosylation (b) with a more severe phenotype.	[93]
		Mouse	Knockout	-Incompatible with life.	[75]
**Protein *N*-glycosylation**	PMM2-CDG/Phosphomannomutase 2	Mouse	(a) Homozygous Pmm2^R137H/R137H^ (b) Homozygous Pmm2^F118L/F118L^ (c) Heterozygous Pmm2^R137H/F118L^	(a) Embryonic lethality. (b) Viable with no phenotype. (c) Embryonic lethality recovered by oral Man administration to pregnant dams.	[77]
		Mouse	Heterozygous Pmm2^R137H/F115L^	-Prenatal lethality which could not be restored with Man supplementation. -Survival mice presented delayed growth and impaired general protein glycosylation.	[94]
		Zebrafish	*Pmm2* knockout	-Reduced Pmm2 enzymatic activity, decreased LLO levels and Man-6-P accumulation. -Impaired motility and altered craniofacial cartilage development.	[95]
		*Drosophila melanogaster*	*Pmm2*-null mutant using CRISPR/Cas9	-Reduced lifespan, psychomotor retardation and impairment of the synaptic matrix metalloproteinase (MMP) pathway.	[96]
		*D. melanogaster*	*Pmm2* knockdown adults using RNAi	-Severe ataxia, loss of coordination and inability to fly.	[96]
**Multiple and other glycosylation pathways**	ATP6AP1-CDG/Accessory subunit of the vacuolar (V)-ATPase protein pump	*S. cerevisiae*	*voa1::H vma21QQ* strain	-Voa1 is the yeast homologue for human ATPase H^+^ transporting accessory protein 1 (ATP6AP1). -E356K and Y313C mutations compromise cell growth.	[97]
		Zebrafish	Homozygous *Atp6ap1b^a82/a82^*	-Pigmentation defects.	[98]
**Multiple and other glycosylation pathways**	ATP6AP1-CDG/Accessory subunit of the vacuolar (V)-ATPase protein pump	Zebrafish	(a) Zygotic *Atp6ap1b* knockdown (b) Maternal and zygotic *Atp6ap1b* knockdown	(a) Eye abnormalities at 3–5 days of development. (b) Reduced development of precursor cells (DFCs) which resulted in smaller Kupffer’s vesicle (KV) organ size, due to reduced KV cell number. Defects in the development of ciliated organs, *spaw* and heart laterality defects were also observed. Loss of Atp6ap1b led to V-ATPase mislocalization and affected DFCs pH.	[98]
		Mouse	Chimeric model with reduced Atp6ap1 (ac45) expression	-One chimeric female that died approximately 6 weeks after birth.	[99]
	CAD-CDG/Enzyme complex (ATase, CPSase, ATCase and DHOase)	CHO cell line	CHO-G9C CAD-deficient cells	-Reduced levels of uridine diphosphate- *N*-acetylglucosamine (UDP-GlcNAc), UDP-*N*-acetylgalactosamine (UDP-GalNAc), UDP-galactose (UDP-Gal), UDP-glucose (UDP-Glc), uridine triphosphate (UTP) and cytosine triphosphate (CTP) (restored by addition of exogenous uridine) as well as less aspartate incorporation into nucleic acids which led to defective growth.	[100]
		*Caenorhabditis elegans*	*pyr-1(cu8)* mutant	-Maternal effect (mother environment and genotype influence) lethality. -Defective pharynx development. Cytoskeletal organization defects.	[101]
		*D. melanogaster*	*Rudimentary* mutant	-High sterility levels in homozygous females. -Reduced viability and defective wing morphogenesis. -Increased survival after uridine, orotic acid, free uracil and carbamoyl aspartic acid supplementation.	[102]
**Multiple and other glycosylation pathways**	CAD-CDG/Enzyme complex (ATase, CPSase, ATCase and DHOase)	Zebrafish	*Perplexed* (*plx^a52^*) mutant	-Impaired retinal, tectal fin and jaw morphogenesis. -Phenotype rescue by pyrimidine treatment.	[103]
		Zebrafish	Transgenic *Tg*(*p2xr3.2:gfp*)^*sl23*^ mutant	-Cranial sensory circuit malformation. -Small eyes and deformed jaws. -Orotic acid or uridine treatment failed to rescue phenotype.	[104]
	CCDC115-CDG/Coiled-coil domain-containing protein 115	*S. cerevisiae*	HY13 (vma22∆::LEU2) KHY34 (vma22∆::LEU2 pep4-3) KHY38 (vma22∆::URA3) KHY39 (vma22∆::URA3 pep4-3)	-No vacuolar ATPase activity. -Normal production of V_1_ subunits, but lack of association to the vacuolar membrane. -Destabilization of Vph1p and Vma3p/Vma11p subunits.	[105]
		HeLa cell line	*CCDC115* knockdown using CRISPR/Cas9	-Impaired iron(II) prolyl hydroxylase (PHD) activity and hypoxia-inducible factor 1 (HIF1α) activation.	[106]
	DOLK-CDG/Dolichol kinase	*S. cerevisiae*	*Sec59* mutant	-Sec59, the homologue of human dolichol kinase (DOLK), catalyzes the phosphorylation of the dolichol lipid carrier. -Thermosensitive.	[18,29]
**Multiple and other glycosylation pathways**	GNE-CDG/UDP-GlcNAc 2-epimerase/ManNAc kinase	CHO cell line	*Gne*-deficient *lec3* mutant	-Reduced NCAM polysialic acid content. -Lec3 cells are defective in GNE activity. -Sialylation defects were rescued by ManNAc and manosamine complementation. -Expression of *Gne* mutation M712T is responsible for less celular and glycoprotein-linked sialic acid content (HIBM) whereas R-236L and R266Q produce higher amounts than WT (sialuria).	[19,26,28,107,108]
		HEK293 cell line	(a) D176V-*Gne* mutant (b) V572L-*Gne* mutant (c) *Gne* knockdown by shRNA	-(a), (b) and (c) have decreasingly levels of sialylation of membrane and cytosolic protein, restored by supplementation with Neu5Ac (SA) and ManNAc. -β-integrin hyposialylation leads to increased cell adhesion.	[39,109]
		Human promyelocytic leukemia (HL60) cell line	HL60-I clone	-Increased overall surface SA content by supplementation with ManNAc and ManNProp.	[110,111]
		*Spodoptera frugiperda* (Sf9) cell line	M712T *Gne*	-Reduced activity but not overall sialylation, indicating that disease is not directly caused by lack of SA.	[40]
		Embryonic stem cells (ESC)	Mice *GNE*^−/−^ ESC	-Impaired proliferation, differentiation and altered genetic expression. -Smaller embryonic bodies size which was corrected by the presence of SA.	[74,112,113,114,115]
**Multiple and other glycosylation pathways**	GNE-CDG/UDP-GlcNAc 2-epimerase/ ManNAc kinase	Mouse	*Gne* ^−/−^	-Incompatible with life.	[74]
		Mouse	*Gne* ^−/+^	-No disease phenotype.	[116]
		Mouse	*Gne*^M712T/M712T^ in a C57BL/6J background	-High lethality within the first 72 h after birth, which was prevented by oral administration of ManNAc to the pregnant and nursing dams. -Muscle hyposialylation at an adult (4 to 6 months) age. -Renal phenotype.	[81,117]
		Mouse	*Gne*^M712T/M712T^ in a mixed genetic background (129Sv/ICR)	-High survival rate. -No renal phenotype. -No muscle deterioration up to 18 months of age.	[76]
		Mouse	Transgenic mouse (Gne^(−/−)^h*GNE*D176V-Tg)	-Decreased levels of SA in different organs. -Adult onset with muscle pathology. -β-amyloid deposits in the muscles, but not in the CNS.	[118,119]
		Mouse	*Gne* ^V572L/V572L^	-Renal phenotype that was corrected by SA administration.	[79]
		Mouse	Transgenic FVBN-GNR-R263L	-Sialuria with elevated SA in urine. -Increased neural cell adhesion molecule (NCAM) polysialylation. -Increased cell surface sialylation in leucocytes.	[120]
**Multiple and other glycosylation pathways**	GNE-CDG/UDP-GlcNAc 2-epimerase/ManNAc kinase	Zebrafish	*Gne* knockout	-High mortality levels. -Impaired muscle structure and development with consequent decreased locomotor activity. -Slightly reduced GNE enzymatic activity.	[121]
	NANS-CDG/CMP-*N*-acetylneuraminic acid synthetase	Zebrafish	*Nansa* and *Nansb* knockdown	*-Nansb* disruption did not generated a disease phenotype. *-Nansa* morphant embryos displayed a small head with a complex phenotype, pericardial edema and skeletal developmental impairment. -Addition of SA to the zebrafish water resulted in partial rescue of the skeletal phenotype but only if added 24 hpf.	[122]
	PGM1-CDG/Phosphogluco-mutase 1	Hela cell line	*Pgm1* and *LDB3* two hybrid system	-DC-related *LDB3* mutations impair binding of PGM1 to Z-band alternatively spliced PDZ-motif (ZASP)/Cypher. -PGM/PMM domain IV of PGM1 is essential for recruitment of ZASP/Cypher.	[123]
	PGM3-CDG/Phosphogluco-mutase 3	Mouse	(a) Hypomorphic (*Pgm3^mld1^*) (b) Null (*Pgm3^gt^*)	(a) Embryonic lethality. (b) Reduced viability with no major alteration in general protein glycosylation, except for the testis-specific isoform of angiotensin-converting enzyme (ACE). Reduced size, mild anemia, splenomegaly, thrombocytopenia, glomerulonephritis and low B- and T-cell numbers.	[124]
**Multiple and other glycosylation pathways**	SLC35A1-CDG/CMP-sialic acid transporter	CHO cell line	*Lec2 (Slc35a1)* mutants	-Asialo phenotype at the cell membrane and unable to translocate CMP-SA to the lumen of the Golgi. -When combined with large overexpression, the α-DG is functionally glycosylated.	[17,25,26,38,125]
		CHO cell line	MAR-11 mutant	-Decreased levels of surface SA.	[126]
		Near-haploid human (HAP1) cell line	*Slc35a1* knockout using transcription activator-like effector nucleases (TALEN)	-SLC35A1 is required for α-DG mannosylation, independently from sialylation.	[32]
	SLC35A2-CDG/UDP-galactose transporter	CHO cell line	*Lec8(Slc35a2)* deficient	-Defective galactosylation. -Gal treatment slightly increased galactosylation.	[33]
		MDCK-RCA^r^ cell line	*Slc35a2* deficient	-Defective galactosylation. -UGT1 and UGT2 are localized in the Golgi the ER, respectively. -UGT forms complexes with NGT and Mgats.	[20,21,33]
		*C. elegans*	*Srf-3* mutants	-Reduced O- and N-linked glycans.	[127]
	SCL35C1-CDG/GDP-fucose transporter	CHO cell line	*Slc35c1* knockout (CHO-gmt5) derived from MAR-11 mutants.	-Asialylated and afucosylated proteins due to absence of functional CMP-sialic acid and GDP-fucose transporter.	[128,129]
		CHO cell line	*Slc35c1* disruption by zinc fingers, TALEN and CRISPR (CHO-gmt3)	-Lack of functional GDP-fucose transporter. -Fucose free glycoproteins.	[130]
		ESC cell line	*Slc35c1* knockout	-Abolishment of *N*- and *O*-glycoproteins fucosylation. -Ricin resistant.	[131]
**Multiple and other glycosylation pathways**	SCL35C1-CDG/GDP-fucose transporter	Mouse	Slc35c1 (*Slc35c1*^−/−^) knockout	-Elevated postnatal mortality, severe growth retardation and immune system affectation. -Significant reduction of fucosylated selectin ligans as well as severe impairment of P-, E- and L-selectin ligand function. -Defective neutrophil migration to the inflamed peritoneum, reduced leukocyte rolling to inflamed muscle venules and absent lymphocyte homing to lymph nodes but normal homing of lymphocytes to the spleen. Normal very long chain polyunsaturated fatty acids (VLC-PUFAs) levels.	[132,133,134]
		Zebrafish	*Slytherin* (*srn*) mutant with a point mutation	-Bent tail that became progressively more severe. -Reduced protein fucosylation in CNS and other tissues. -Reduced number of neurons and glia cells. -Signaling reduction in the Notch-Delta pathway leading to defective neuromuscular synaptogenesis.	[135,136]
		Zebrafish	Slc35c1 protein over-expression	-Increased N-linked fucosylation and disruption of embryonic patterning. -Negative regulation of Wnt signaling.	[137]
	SLC39A8-CDG/Solute carrier family 39 (zinc transporter), member 8—ZIP8	Mouse	*Slc39a8*^−/−^ knockout	-Impaired cardiovascular function, absence of sternum, small chest cavity and a small liver.	[85]
		Mouse	Hypomorphic *Slc39a8*^(neo/neo)^	-Reduced mRNA and protein levels of the ZIP8 Zn^2+^/(HCO_3_^-^)_2_ symporter in several tissues of the neonate mutants. -Reduced zinc and iron levels. -Embryonic and neonatal lethality. -Surviving offspring was pale, presented growth arrest, severe anemia, hypoplastic spleen, hypoplasia of liver, kidney, lung and lower extremities.	[138,139]
	SRD5A3-CDG/Steroid 5 α-reductase 3	*S. cerevisiae*	*Dfg10-100*	-SRD5A3 is the human ortholog of yeast dfg10. -Defective filamentous growth and carboxypeptidase Y (CPY) hypoglycosylation. -Defective metabolism of polyprenol to dolichol.	[140]
**Multiple and other glycosylation pathways**	SRD5A3-CDG/Steroid 5 α-reductase 3	Mouse	Homozygous (*Srd5a3^Gt/Gt^*)	-Complete embryonic lethality beyond E12.5. -Homozygous embryos were smaller and displayed dilated hearts and open neural tubes. -Transcriptomic analysis revealed an up-regulation of the unfolded protein response (UPR) and a downregulation of genes involved in general cellular metabolic processes and specific embryonic development. -Elevated polyprenol levels.	[140]
	TMEM165-CDG/Transmembrane protein 165	*S. cerevisiae*	*Gdt1∆*	-Growth defect and defective glycosylation in high Ca^2+^ concentration which are supressed by Mn^2+^ administration. -Gdt1p controls cellular calcium stores and respond to osmotic shock.	[30,141,142]
		HEK293 cell line	*TMEM165* knockout by CRISPR	-TMEM165 degradation in lysosomes upon Mn^2+^ exposure.	[36]
		HEK293 cell line	*TMEM165* knockdown by shRNA	-Impaired Golgi Mn^2+^ homeostasis. -Mn^2+^ rescues impaired Golgi glycosylation.	[142]
		HeLa cell line	*TMEM165* knockdown by shRNA	-Impaired Golgi Mn^2+^ homeostasis.	[142]
**Multiple and other glycosylation pathways**	TMEM165-CDG/Transmembrane protein 165	Zebrafish	Homozygous null *tmem165* (*tmem165*^−/−^)	-Dysfunctional *N*-glycosylation, reduced osteoblast differentiation and altered craniofacial cartilage development due to defects in chondrocyte maturation.	[143]
	TMEM199-CDG/Transmembrane protein 199	*S. cerevisiae*	DJY62/DJY102 (pep4–3 vma12∆::LEU2) DJY63 (vma12∆::LEU2)	-Decreased stability of 100-kDa V_0_ subunit (Vph1p) of V-ATPase.	[105,144]
		HeLa cell line	*TMEM199* knockdown by CRISPR/Cas9	-Lethality after three weeks and accumulation of HIF1α.	[106]
**Lipid glycosylation and glycosylphosphatidyl inositol (GPI) synthesis**	PIGA-CDG/Phosphatidylinositol *N*-acetylglucosaminyl-transferase (subunit A)	iPSC	(a) Hypomorphic (PIGAc.1234C>T) (b) *PIGA* null	(a) Permissive for hematopoiesis with neuronal proliferation, differentiation, maturation and presynaptic defects. (b) Non-permissive for hematopoiesis and differentiation.	[145]
		Mouse	*Piga*-deficient chimeric mice	-Chimeric surface expression of GPI-anchored proteins.	[146]
		Mouse	Partial exon 2 excision mediated by loxP	-Viable mosaic mice with lack of GPI-linked proteins on a proportion of circulating blood cells. -Increased sensitivity toward complement mediated lysis and a decreased life span in circulation.	[147]
	PIGM-CDG/GPI α-1,4-mannosyltransferase I	Ramos517 cell line	PIGM-deficient	-Cloning of human homologues PfPIG-M (*Plasmodium falciparum*) and GPI14 (*S. cerevisiae*) but only PfPIG-M restored cell-surface expression of GPI proteins.	[148]
	PIGM-CDG/GPI α-1,4-mannosyltransferase I	*S. cerevisiae*	*Gpi14* (*PIGM* homolog) -deficient	-Glucosaminyl(acyl)phosphatidylinositol accumulation. -Growth lethality.	[149]
**Lipid glycosylation and GPI synthesis**	PIGO-CDG/GPI ethanolamine phosphate transferase 3	CHO cell line	*PIGO*-deficient cells	-Impaired levels of CD59 and urokinase receptor (uPAR).	[35,37]
		HEK293 cell line	*PIGO* knockout using CRISPR/Cas9	-Impaired GPI-AP expression.	[35]
***O*-mannosyl-glycan synthesis**	ISPD-CDG/2-C-methyl-d-erythritol 4-phosphate cytidylyltransferase	HEK293 cell line	*Ispd* knockout using CRISPR/Cas9	-Reduced α-DG glycosylation. -ISPD synthesize CDP-ribitol required for α-DG glycosylation.	[68]
		HAP1 cell line	*Ispd*-disrupted cells by a CRISPR/Cas9 deletion causing a frameshift	-Supplementation with CDP-Rbo restored α-DG glycosylation.	[150]
		Mouse	Homozygous *Ispd*^L79*/L79*^	-No embryonic lethality, but did not survived beyond birth due to apparent respiratory failure. -Normal total dystroglycan protein levels, but severely reduced levels of α-DG and laminin binding activity in brain extracts. -Lose of dystroglycan glycosylation in cortex extracts.	[151]
***O*-mannosyl-glycan synthesis**	ISPD-CDG/2-C-methyl-d-erythritol 4-phosphate cytidylyltransferase	Mouse	*Ispd* conditional knockout using Cas9 nickase (Cas9n) and a single guide RNA (sgRNA)	-No phenotype available.	[152]
		Zebrafish	*Ispd* knock-out	-Incomplete brain folding in the majority of the embryos as well as hydrocephalus, reduced eye size, muscle fiber degeneration and impaired motility. -Protein hypoglycosylation, especially for α-DG.	[153]

**Table 2:** Overview of diagnosis biomarkers reported for CDG.

CDG/Protein	Biomarker	Sample *	Major Findings	Detection Technique	Reference
**CDG-I/CDG-II**	Transferrin	Serum/plasma	Altered glycosylation pattern	IEF, high performance liquid chromatography (HPLC), capilary zone electrophoresis (CZE)	[7,156]
**CDG-I/CDG-II**	α_1_-antitrypsin	Serum/plasma	Altered glycosylation pattern	2-Dimensional difference gel electrophoresis (2D DIGE), IEF	[156]
**CDG-II**	Lipoprotein ApoCIII	Serum/plasma	Altered profile of the three protein isoforms: apoCIII_0_, apoCIII_1_ and apoCIII_2_ (hypoglycosylation)	IEF	[4,7,157]
**ALG1-CDG/β-1,4-mannosyl-transferase**	Tetrasaccharide (NeuAc-Gal-GlcNAc2)	Serum/plasma TF	Increased levels	Liquid chromatography—mass spectrometry (LC/MS) and enzymatic digestions	[34,176]
**ALG6-CDG/Glucosyl-Transferase**	Man_9_GlcNAc_2_-P-P-dolichol	Fibroblasts	Increased levels	2-[^3^H]mannose labeling and HPLC analysis	[177]
**ALG1-CDG/β-1,4-mannosyl-transferase, MPI-CDG/Phospho-mannose isomerase, PMM2-CDG/Phosphomannomutase 2**	*N*-tetrasaccharide (Neu_5_Ac_2,6Gal_1,4-GlcNAc_1,4GlcNAc)	Sera, plasma and fibroblasts	Increased levels compared to control	LC-MS/MS Matrix assisted laser desorption/ionization-time of flight-mass spectrometry (MALDI-TOF-MS)	[178]

* Unless otherwise specified, the samples are derived from patients.

**Table 3:** Overview of other biomarkers reported for CDG.

CDG/Protein	Biomarker	Sample *	Major Findings	Detection Technique	Reference
**CDG-I**	Glyc-ER-GFP	Fibroblasts and iPSCs	Fluorescence	Flow Cytometry	[179]
**CDG-I**	Thyroxine binding globulin	Serum	Abnormal glycosylation	IEF	[180]
**CDG-II**	Glyc-ER-GFP	Fibroblasts	No fluorescence	Flow Cytometry	[179]
**CDG-II**	(a) α_1_-acid glycoprotein (b) Ceruloplasmin	Serum	(a) Extra isoform with higher pI value (b) Subtle pI change	2D DIGE	[155]
**GNE-CDG/UDP-GlcNAc 2-epimerase/ManNAc kinase**	GM3 and GD3 gangliosides	Human embryonic kidney (HEK AD293) cells Muscle of Gne^M712T/M712T^ mouse model	Increased levels	Flow cytometry HPLC	[181,182]
	NCAM	Brain solubilisates from heterozygous GNE-deficient mice Serum obtained from patients and Gne^M712T/M712T^ mouse	Hypolysialylated	WB	[116,183]
	Thomsen-Friedenreich (T)-antigen	Plasma	Increased ratio of T-antigen (Gal-GalNAc-) to ST (sialylated)- antigen (core 1 SA-Gal-GalNAc-)	MS	[184]
**PMM2-CDG/Phosphomannomutase 2**	Band 3 and glycophorin A	Erythrocytes	Underglycosylated	SDS-PAGE	[185]
	Glycosphingolipids (Gb3, GM2, GD3 and GD1a)	Fibroblasts	Increased levels compared to control	Radiolabeling followed by HPTLC	[186]
**PMM2-CDG/Phosphomannomutase 2**	(a) α_1_-acid glycoprotein (b) Ceruloplasmin (c) α_1_-antichymotrypsin (d) α_1_B-glycoprotein (e) Haptoglobin	Serum	(a) Lower MW isoform (b) Profound pI change (c) Abnormal profile (d) Abnormal profile (e) Absence of stainable protein	2D DIGE	[155,187]
	TSH and TF_4_	Serum	Increased TSH and decreased TF_4_ levels	Immune-based method	[4,188]
**ALG6-CDG/Glucosyl-Transferase, PMM2-CDG/Phosphomannomutase 2**	β-trace protein	Cerebrospinal fluid	Abnormal glycosylation profile	SDS-PAGE and immunoblotting	[180,189]
**ALG6-CDG/Glucosyl-Transferase, MPI-CDG/Phospho-mannose isomerase, PMM2-CDG/Phosphomannomutase 2**	Aspartylglucosaminidase (AGA)	Plasma	Increased levels	Enzymatic activity	[166]
**ALG13-CDG/UDP-GlcNAc transferase, MPI-CDG/Phospho-mannose isomerase, PMM2-CDG/Phosphomannomutase 2**	ICAM-1	(a) Lec9 CHO cells and fibroblasts (b) Mesenteric endothelial cells of Mpi^−/−^ mouse	Decreased levels	(a) LC-MS/MS SDS-PAGE and WB IF staining Flow Cytometry (b) Immuno- histochemistry	[174,175,190]

* Unless otherwise specified, the samples are derived from patients. Glyc-ER-GFP—*N*-glycosylated-endoplasmic reticulum targeted-green fluorescent protein, TF—Transferrin, CZE—capilary zone electrophoresis, LC-MS/MS—Liquid chromatography—tandem mass spectrometry, MALDI-TOF-MS—Matrix assisted laser desorption/ionization-time of flight-mass spectrometry, HPLC—High performance liquid chromatography, SDS-PAGE—Sodium odecyl sulfate polyacrylamide gel electrophoresis, Gb3—globotrihexosylceramide, GM2—Ganglioside monosialic 2, GD3—Ganglioside disialic 3, GD1a—Ganglioside disialic 1a, HPTLC—High-performance thin-layer chromatography, VCAM—Neural cell adhesion molecule, IEF—Isoelectric focusing, GM3—Ganglioside monosialic 3, ICAM—Intercellular cell adhesion molecule, , WB—Western blotting, IF—Immunofluorescence, 2D DIGE—2-Dimensional difference gel electrophoresis, MW—Molecular weight, pI—Isoelectric point, AT-III—antithrombin III, TSH—Thyroid stimulating hormone, TF_4_—Thyroxine.

**Table 4:** Summary of the clinical trials registered at ClinicalTrials.gov and ClinicalTrialsregister.eu, related to Congenital Disorders of Glycosylation. The results were obtained using the following keywords: Congenital Disorders of Glycosylation, GNE, PMM2-CDG and Cerebellar Disease.

Study Identifier	Status	Study Title	Condition	Intervention	Study Characteristics	Study Type
**NCT02089789** ^§^	Active, recruiting	Clinical and basic investigations into known and suspected Congenital Disorders of Glycosylation	CDG	N.A	N.A	Observational
**NCT02503267** ^§^	Active, recruiting	Incidence and consequences of Disorders of Glycosylation in patients with conotruncal and septal heart defects (CARDIoG)	CDG	N.A	N.A	Observational
**NCT03250728** ^§^	Active, not recruiting	Role of the endothelium in stroke-like episode among CDG Patients (PECDG)	CDG	N.A	N.A	Interventional Peripheral blood puncture
**NCT02955264**^§^ [208]	Active, recruiting	Using d-Galactose as a food supplement in Congenital Disorders of Glycosylation	CDG	**Drug**: d-galactose (dietary supplement) **Administration**: Oral	Open label, single group	Interventional Phase 2
**NCT02346461** ^§^	Active, not recruiting	An open label Phase 2 study of ManNAc in subjects with GNE Myopathy	-GNE-CDG	**Drug**: ManNAc **Administration**: Oral	Open label, Non-randomized	Interventional Phase 2
**NCT01634750**^§^ [264]	Completed	Phase I clinical trial of ManNAc in patients with GNE Myopathy or Hereditary Inclusion Body Myopathy (HIBM)	-GNE-CDG -Hereditary Inclusion Body Myopathy (HIBM)	**Drug**: ManNAc **Administration**: Oral	Randomized, double-blind, placebo-controlled	Interventional Phase 1
**NCT02736188**^§∞^ **2016-000360-42** *^∞^	Active, not recruiting	Study to evaluate the safety and efficacy of Ace-ER Tablets in patients with GNE Myopathy or Hereditary Inclusion Body Myopathy	-GNE-CDG -HIBM -Quadriceps Sparing Myopathy -Distal Myopathy With Rimmed Vacuoles	**Drug**: Aceneuramic Acid Extended-Release Tablets (Ace-ER) **Administration**: Oral	Open label, single group	Interventional Phase 3
**NCT02731690**^§≈^ **2015-004553-41** *^≈^	Active, not recruiting	A study to evaluate the safety of Aceneuramic Acid Extended Release (Ace-ER) tablets in GNE Myopathy (GNEM) (Also Known as Hereditary Inclusion Body Myopathy (HIBM)) patients with severe ambulatory impairment	-GNE-CDG	**Drug**: Aceneuramic Acid Extended-Release tablets (Ace-ER) **Administration**: Oral	Open label, single group	Interventional Phase 2
**NCT01517880**^§^ [265]	Completed	A phase 2 study to evaluate the dose and pharmacodynamic efficacy of Sialic Acid-Extended Release (SA-ER) tablets in patients with GNE Myopathy or Hereditary Inclusion Body Myopathy (HIBM)	-GNE-CDG -HIBM	**Drug**: Sialic Acid Extended Release (SA-ER) **Administration**: Oral	Randomized, double-blind, placebo-controlled	Interventional Phase 2
**NCT01830972** ^§^	Completed	An open label phase 2 extension study of higher dose Sialic Acid (ER Tablets + IR Capsules) in patients with GNE Myopathy	-GNE-CDG	**Drug**: Sialic Acid Extended Release (SA-ER) Sialic Acid Immediate Release (SA-IR) **Administration**: Oral	Open label, non-randomized	Interventional Phase 2
**NCT01359319**^§^ [265]	Completed	Safety and pharmacokinetics of Sialic Acid tables in patients With Hereditary Inclusion Body Myopathy (HIBM)	-GNE-CDG -HIBM	**Drug**: Sialic Acid Extended Release (SA-ER) tablets **Administration**: Oral	Open label, non-randomized, single group	Interventional Phase 1
**NCT02377921** ^§^	Completed	Phase 3 randomized, double-blind, placebo-controlled study to evaluate Sialic Acid in patients with GNE Myopathy or Hereditary Inclusion Body Myopathy (HIBM)	-GNECDG -HIBM	**Drug**: Sialic Acid Tablets (UX001) **Administration**: Oral	Randomized, double-blind, placebo-controlled	Interventional Phase 3
**NCT00195637**^§^ [266]	Completed	Intravenous immune globulin to treat Hereditary Inclusion Body Myopathy	-GNE-CDG -HIBM	**Drug**: Immune Globulin **Administration**: intravenous	Pilot study with 4 participants	Interventional Phase 1
**NCT01236898** ^§^	Completed	Pharmacokinetic study on *N*-acetylneuraminic Acid	-GNE-CDG -HIBM	**Drug**: *N*-acetylneuraminic acid (anhydride) (NPC-09) **Administration**: Oral	Open label, non-randomized, single group	Interventional Phase 1
**NCT01784679** ^§^	Active, recruiting	GNE-Myopathy disease monitoring program (GNEM-DMP): a registry and prospective observational natural history study to assess GNE Myopathy or Hereditary Inclusion Body Myopathy (HIBM)	-GNE-CDG -HIBM	N.A	N.A	Observational
**NCT02196909** ^§^	Active, not recruiting	Clinical, biological and NMR outcome measures study for Hereditary Inclusion Body Myopathy due to mutation of UDP-*N*-acetylglucosamine 2-epimerase/*N*-acetylmannosamine kinase gene (GNE) (ClinBio-GNE)	-GNE-CDG -HIBM	N.A	N.A	Interventional Parallel assignment with blood and urine collection
**NCT01902940** ^§^	Completed	Natural history in CCFDN and IBM syndromes	-GNE-CDG -HIBM	N.A	N.A	Observational
**NCT01417533** ^§^	Active, recruiting	A natural history study of patients with GNE Myopathy	-GNE-CDG -HIBM	N.A	N.A	Observational
**NCT03173300** ^§^	Active, recruiting	Natural history study protocol in PMM2-CDG (CDG-Ia)	PMM2-CDG	N.A	N.A	Observational
**2017-000810-44** *	Active	Phase II clinical trial to evaluate the effectiveness and safety of acetazolamide in the treatment of cerebellar syndrome in patients with PMM2-CDG deficiency	PMM2-CDG	**Drug**: Acetazolamide **Administration**: Oral	Randomized, open labeled	Interventional Phase 2

^§^ Accession number for ClinicalTrials.gov [267]; * Accession number for ClinicalTrialsregister.eu [268]; ^∞^ Despite the current status, this trial has been stopped; ≈ Despite the current status, this trial has been closed; N.A.—Not applied.

## Supplementary Materials

Supplementary materials can be found at http://www.mdpi.com/1422-0067/19/5/1304/s1.

## Abbreviations

α–DG	α-Dystroglycan
AAV	Adeno-associated virus
Ac_4_ManNAc	Peracetylated *N*-acetylmannosamine
ACE	Angiotensin-converting enzyme
AGA	Aspartylglucosaminidase
AMO	Antisense morpholino oligonucleotides
ApoC-III	Apolipoprotein C-III
ATase	Amidophosphoribosyltransferase
ATCase	Aspartate transcarbamoylase
BBB	Blood brain barrier
CCFDN	Congenital cataracts-facial dysmorphism-neuropathy syndrome
CDG	Congenital disorder(s) of glycosylation
CDP	Cytidine diphosphate
CHO	Chinese hamster ovary
CMP	Cytidine monophosphate
CNS	Central nervous system
CPSase	Carbamoyl phosphate synthetase
CPY	Carboxypeptidase Y
CTP	Cytosine triphosphate
DC	Dilated cardiomyopathy
DFCs	Dorsal forerunner cells
DHOase	Dihydroorotase
DIGE	2D-Differential gel electrophoresis
ER	Endoplasmic reticulum
FKTN	Fukutin
FKRP	Fukutin related protein
Fuc	Fucose
GABA	γ-Amino butyric acid
Gal	Galactose
GalNAc	*N*-Acetylgalactosamine
GALE	UDP-galactose-4-epimerase
GALK	Galactokinase
GALT	Galactose-1-phosphate uridyl transferase
GDP	Guanosine diphosphate
Glc	Glucose
GlcNAc	*N*-Acetylglucosamine
GPI	Glycosylphosphatidylinositol
GSL	Glycosphingolipid
HDAC	Histone deacetylase inhibitors
HIBM	Hereditary inclusion body myopathy
HIFs	Hypoxia inducible factors
HIF1α	Hypoxia inducible factor 1-alpha
IBM	Inclusion body myopathy
ICAM-1	Intercellular adhesion molecule 1
IEM	Inborn errors of metabolism
IM	Intramuscular
IMD	Inherited metabolic disease
iPSCs	Induced pluripotent stem cells
IV	Intravenous
KV	Kupffer’s vesicle
LLO	Lipid-linked oligosaccharides
Magts	Mannoside acetylglucosaminyltransferases
Man	Mannose
Man-1-P	Mannose-1-phosphate
Man-6-P	Mannose-6-phosphate
ManN	d-mannosamine
ManNAc	*N*-acetylmannosamine
MCAHS2	Multiple congenital anomalies-hypotonia-seizures syndrome-2
MPG1	Mannose-1-phosphate guanylyltransferase
MPI	Phosphomannose isomerase
MPP	Matrix metalloproteinase
MS	Mass spectrometry
NCAM	Neural cell adhesion molecule
NGT	UDP-*N*-acetylglucosamine transporter
PI	Phosphatidylinositol
PC	Pharmacological chaperone
PMM2	Phosphomannomutase 2
PUFA	Polyunsaturated fatty acids
Rbo	Ribitol
SA	Sialic acid
TF_4_	Thyroxine
TS	*trans*-Splicing
TSH	Thyroid stimulating hormone
UDP	Uridine diphosphate
UGT	UDP-galactose transporter
UMPS	Uridine monophosphate synthetase
uPAR	Urokinase plasminogen activator receptor
UPR	Unfolded protein response
UTP	Uridine triphosphate
VLC-PUFA	Very long chain polyunsaturated fatty acids
Vma3p	17-kDa Proteolipid subunit of vacuolar ATPase
Vma11p	V-Type proton ATPase 16 kDa proteolipid subunit 2
Vph1p	100 kDa Subunit a of vacuolar-ATPase V_0_ domain
XMEN	X-linked immunodeficiency with magnesium defect, EBV infections and neoplasia
