# Supplementary material for: CDG Therapies: From Bench to Bedside
Source: Int J Mol Sci. 2018 Apr 27;19(5):1304. doi: 10.3390/ijms19051304 (PMC5983582; doi:10.3390/ijms19051304)
Supplement: Supplementary file 1 [file ijms-19-01304-s001.pdf]

Supplementary Table 1 - Overview of the ongoing clinical trials (CTs), available CDG *in vitro* and *in vivo* models and biomarkers and dietary supplementation strategies, being tested and used in these disorders. NA – Not available.

	**ALG1-CDG Chitobiosyldiphospho-dolichol β-mannosyltransferase**	**ALG6-CDG α-1,3-glucosyltransferase**	**ALG13-CDG UDP-GlcNAc transferase**	**ATP6AP1-CDG Accessory subunit of the vacuolar (V)-ATPase protein pump**	**CAD-CDG Tri-functional protein (ATase, CPSase, ATCase and DHOase)**	**CCDC115-CDG Coiled-coil domain-Containing protein 115**
**CTs**	Interventional NCT02955264	Interventional NCT02955264	NA		NA	NA
***In vitro* models**	*S. cerevisiae alg1* mutant (K57-6C strain) [24,34,41]	*S. cerevisiae alg6* mutant [87,88]	*S. cerevisiae* “Tet-Off “ strain (pYGL047W::kanR-tet07-TATA URA3::CMV-tTA MATa his3-1 leu2-0 met15-0) [281]	*S. cerevisiae* (*voa1::H vma21QQ* strain) [97]	*CAD*-deficient CHO-G9C [100]	*S. cerevisiae* strains: HY13 (vma22Δ::LEU2) KHY34 (vma22Δ::LEU2 pep4-3) KHY38 (vma22Δ::URA3) KHY39 (vma22Δ::URA3 pep4-3) [105]
	S. cerevisiae alg1 mutant (PRY56 strain) [31]	MI8-5 *Alg6*$^{-/-}$ [89]				HeLa (*CCDC115* knockdown using CRISPR/Cas9) [106]
***In vivo* models**	NA	NA	NA	Zebrafish (*Atp6ap1b*$^{a82/a82}$,*Atp6ap1b* *k*nockdown) [98]	*C. elegans pyr-1(cu8)* mutant [85]	NA
					D. melanogaster Rudimentary mutant [86]	

Supplementary Table 2 – continued.

	**ALG1-CDG Chitobiosyldiphospho-dolichol β-mannosyltransferase**	**ALG6-CDG α-1,3-glucosyltransferase**	**ALG13-CDG UDP-GlcNAc transferase**	**ATP6AP1-CDG Accessory subunit of the vacuolar (V)-ATPase protein pump**	**CAD-CDG Tri-functional protein (ATase, CPSase, ATCase and DHOase)**	**CCDC115-CDG Coiled-coil domain-Containing protein 115**
***In vivo* models**	NA	NA	NA	Chimeric Mouse (w/ *Atp6ap1* reduced expression) [99]	Zebrafish *Perplexed* (*plx*^{a52}) mutant [87]<br>Zebrafish Transgenic *Tg(p2xr3.2:gfp)*^{sl23} mutant [88]	NA
**Biomarkers**	$GlcNAc_2$-PP-dolichol [31]<br>N-tetrasaccharide (Neu_5Ac_2,6Gal_1,4-GlcNAc_1,4GlcNAc) [34,178]	Dolichol-linked $Man_9GlcNAc_2$ [177]<br>AGA [166]<br>β-trace protein [180,189]	GlcNAc-PP-dolichol<br>ICAM-1 [190]	NA	NA	NA
**Dietery therapy**	Man suppl [178]	NA	Gal suppl [190]	NA	Uridine suppl [69,100]	Iron suppl [106]
**Transplantation**	NA	NA	NA	Liver transplantation (approved therapy in Europe)	NA	Liver transplantation (approved therapy in Europe) [224]

Supplementary Table 3 - continued.

	**ALG1-CDG Chitobiosyldiphospho-dolichol β-mannosyltransferase**	**ALG6-CDG α-1,3-glucosyltransferase**	**ALG13-CDG UDP-GlcNAc transferase**	**ATP6AP1-CDG Accessory subunit of the vacuolar (V)-ATPase protein pump**	**CAD-CDG Tri-functional protein (ATase, CPSase, ATCase and DHOase)**	**CCDC115-CDG Coiled-coil domain-Containing protein 115**
**Gene therapy**	NA	NA	NA	NA	NA	NA
**Antisense therapy**	NA	NA	NA	NA	NA	NA
**PCs**	NA	NA	NA	NA	NA	NA
**Others**	NPCRS [255,256]	NPCRS [255,256]	NPCRS [255,256]	NPCRS [255,256]	NPCRS [255,256]	NPCRS [255,256]

	**DOLK-CDG Dolichol kinase**	**GNE-CDG UDP-GlcNAc 2-epimerase/ ManNAc kinase**	**ISPD-CDG Isoprenoid synthase domain-containing protein**	**MAGT1-CDG Magnesium transporter 1**	**MPI-CDG Mannose-6-phosphate isomerase**	**NANS-CDG CMP-N-acetylneuraminic acid synthetase**
**CTs**	NA	Interventional NCT02346461 NCT01634750 NCT01517880	NA	NA	NA	SA trial approved but discontinued due unfavorable results in GNE-CDG

Supplementary Table 4 - continued.

<table>
<tr><th></th><th>DOLK-CDG
Dolichol kinase</th><th>GNE-CDG
UDP-GlcNAc 2-epimerase/
ManNAc kinase</th><th>ISPD-CDG
Isoprenoid synthase domain-containing protein</th><th>MAGT1-CDG
Magnesium transporter 1</th><th>MPI-CDG
Mannose-6-phosphate isomerase</th><th>NANS-CDG
CMP-N-acetylneuraminic acid synthetase</th></tr>
<tr><td rowspan="2">CTs</td><td rowspan="2">-</td><td>Interventional
NCT01830972
NCT02377921
NCT02736188
NCT02731690
NCT01359319
NCT00195637
NCT01236898
NCT02196909</td><td rowspan="2">-</td><td rowspan="2">-</td><td rowspan="2">-</td><td rowspan="2">-</td></tr>
<tr><td>Observational
NCT01784679
NCT01902940
NCT01417533</td></tr>
<tr><td rowspan="6">In vitro models</td><td rowspan="6">S. cerevisiae Sec59 mutant [18,29]</td><td>CHO Gne-deficient Lec3 mutant [19,26,28,107,108]</td><td rowspan="3">HEK293 Ispd knockout [68]</td><td rowspan="3">S. cerevisiae Alr1Δ strain [90]</td><td rowspan="6">HT-29 Mpi knockdown [59]</td><td rowspan="6">NA</td></tr>
<tr><td>HEK293 (D176V-Gne, V572L-Gne and Gne knockdown) [39,109]</td></tr>
<tr><td>BJA-B K20 (D176V-Gne and M712L-Gne) [110,111,206,207]</td></tr>
<tr><td>HL60-I [110,111]</td><td rowspan="3">HAP1 Ispd knockout [150]</td><td rowspan="3">HEK293 Magt1 knock down [90]</td></tr>
<tr><td>Sf9 M712T-Gne [40]</td></tr>
<tr><td>Gne$^{-/-}$ mice ESC [74,112,113,115]</td></tr>
</table>

Supplementary Table 5 - continued.

	**DOLK-CDG**<br>**Dolichol kinase**	**GNE-CDG**<br>**UDP-GlcNAc 2-epimerase/ ManNAc kinase**	**ISPD-CDG**<br>**Isoprenoid synthase domain-containing protein**	**MAGT1-CDG**<br>**Magnesium transporter 1**	**MPI-CDG**<br>**Mannose-6-phosphate isomerase**	**NANS-CDG**<br>**CMP-N-acetylneuraminic acid synthetase**
***In vivo* models**	NA	Mouse (*Gne*$^{-/-}$, *Gne*$^{+/-}$) [74,116]<br>Mouse (*Gne*$^{M712T/M712T}$) [76,81,117]<br>Mouse (Gne$^{(-/-)}$ h*GNE*D176V-Tg) [118,119]<br>Mouse (*Gne*$^{V572L/V572L}$) [76,81,117]<br>Mouse (Transgenic FVBN-GNR-R263L) [120]<br>Zebrafish (*gne* knock-out) [121]	Mouse (*Ispd*$^{L79*/L79*}$) [151]<br>Mouse (Knock-out) [152]<br>Zebrafish (Knock-out) [153]	Zebrafish (*Magt1* knock-out) [90]<br>NA	Mouse (*Mpi*$^{Y255C/Y255C}$) [72]<br>Mouse (Mpi$^{-/-}$) [91]<br>Zebrafish (w/ 13% of *mpi* activity) [92]	Zebrafish model (*nansa* and *nansb* knockdown) [122]
**Biomarkers**	NA	GM3 and GD3 gangliosides [181,182]<br>NCAM [116,183]<br>Thomsen-Friedenreich (T)-antigen [184]	NA	NA	N-tetrasaccharide (Neu$_5$Ac_2,6Gal_1,4-GlcNAc_1,4GlcNAc [178]<br>AGA [166]<br>ICAM-I [174,175]	NA

Supplementary Table 6 - continued.

	**DOLK-CDG Dolichol kinase**	**GNE-CDG UDP-GlcNAc 2-epimerase/ ManNAc kinase**	**ISPD-CDG Isoprenoid synthase domain-containing protein**	**MAGT1-CDG Magnesium transporter 1**	**MPI-CDG Mannose-6-phosphate isomerase**	**NANS-CDG CMP-N-acetylneuraminic acid synthetase**
**Diatery therapy**	NA	ManNAc suppl [66,80–82,117]<br>ManN suppl [78,83]<br>SA suppl [78,82,83]<br>6′-sialyllactose suppl [82,84]<br>Ac_4ManNAc suppl [83,207]	Ribitol suppl [68,150]	Mg^{2+} suppl [63,215]	Man suppl [49,160–169]	SA suppl [122]
**Transplantation**	Heart transplantation [252,253]	NA	NA	Hematopoietic cell transplantation [215]	Liver transplantation (approved therapy in Europe) [171]	NA
**Gene therapy**	NA	AVV8 [61,243]<br>AVV-TS [244]<br>GNE-lipoplex [28,246–248]	NA	NA	NA	NA

Supplementary Table 7 – continued.

	**DOLK-CDG**<br>**Dolichol kinase**	**GNE-CDG**<br>**UDP-GlcNAc 2-epimerase/ ManNAc kinase**	**ISPD-CDG**<br>**Isoprenoid synthase domain-containing protein**	**MAGT1-CDG**<br>**Magnesium transporter 1**	**MPI-CDG**<br>**Mannose-6-phosphate isomerase**	**NANS-CDG**<br>**CMP-N-acetylneuraminic acid synthetase**
**Antisense therapy**	NA	NA	NA	NA	NA	NA
**PCs**	NA	NA	NA	NA	NA	NA
**Others**	NPCRS [255,256]	NPCRS [255,256]	NPCRS [255,256]<br>GNEM-FAS [261]<br>REMUDY [262,263]	NPCRS [255,256]	NPCRS [255,256]	NPCRS [255,256]

	**PGM1-CDG**<br>**Phosphogluco-mutase 1**	**PGM3-CDG**<br>**Phosphogluco-mutase 3**	**PIGA-CDG**<br>**Phosphatidylinositol N-acetylglucosaminyl-transferase (subunit A)**	**PIGM-CDG**<br>**GPI α-1,4-mannosyltransferase I**	**PIGO-CDG**<br>**GPI ethanolamine phosphate transferase 3**	**PMM2-CDG**<br>**Phosphomannomutase 2**
**CTs**	Interventional<br>NCT02955264	NA	NA	NA	NA	Interventional<br>NCT03250728<br>2017-000810-44<br>Observational<br>NCT03173300
***In vitro* models**	HeLa *Pgm1* and *LDB3* two hybrid system [123]	NA	hiPSC (hypomorphic PIGAc.1234C>T and *Piga* null [145]	Ramos517 cells *Pigm*-deficient [148]<br>*S. cerevisiae* Gpi14-deficient [149]	CHO *Pigo*-deficient [35,37]<br>HEK293 *Pigo* knockout [35]	iPSC (hypomorphic PMM2$^{422G>A/357C>A}$ and PMM2$^{422G>A/357C>A}$-iPSC with additional knockdown [93]

Supplementary Table 8 - continued.

	**PGM1-CDG Phosphoglucomutase 1**	**PGM3-CDG Phosphoglucomutase 3**	**PIGA-CDG Phosphatidylinositol N-acetylglucosaminyl-transferase (subunit A)**	**PIGM-CDG GPI α-1,4-mannosyltransferase I**	**PIGO-CDG GPI ethanolamine phosphate transferase 3**	**PMM2-CDG Phosphomannomutase 2**
***In vivo* models**	NA	Mouse (*Pgm3*^{mld1})<br>[124]<br><br>Mouse (*Pgm3*^{gt})<br>[124]	Chimeric Mouse<br>(*Piga*-deficient)<br>[146]<br><br>Mouse<br>(Partial exon2 excision)<br>[147]	NA	NA	Mouse (Knock-out)<br>[75]<br><br>Mouse (Pmm2$^{R137H/R137H}$)<br>[77]<br><br>Mouse (Pmm2$^{F118L/F118L}$)<br>[77]<br><br>Mouse (Pmm2$^{R137H/F118L}$)<br>[77]<br><br>Mouse (Pmm2$^{R137H/F115L}$)<br>[94]<br><br>Zebrafish (Knock-out)<br>[95]<br><br>Drosophila melanogaster<br>(*pmm2*-null)<br>[96]<br><br>*D.melanogaster*<br>(*pmm2* knockdown)<br>[96]<br><br>*Xenopus laevis*<br>(*Pmm2*-null) [282]
**Biomarkers**	NA	NA	NA	NA	NA	N-tetrasaccharide<br>(Neu5Ac_2,6Gal_1,4-GlcNAc_1,4GlcNAc)<br>[178]

Supplementary Table 9 - continued.

	**PGM1-CDG Phosphogluco-mutase 1**	**PGM3-CDG Phosphogluco-mutase 3**	**PIGA-CDG Phosphatidylinositol N-acetylglucosaminyl-transferase (subunit A)**	**PIGM-CDG GPI α-1,4-mannosyltransferase I**	**PIGO-CDG GPI ethanolamine phosphate transferase 3**	**PMM2-CDG Phosphomanno-mutase 2**
**Biomarkers**	NA	NA	NA	NA	NA	Band 3 and glycophorin A [185]<br>Glycosphingolipids (Gb3, GM2, GD3 and GD1a) [186]<br>AGA [166]<br>ICAM-I [174,175]<br>α_1-acid glycoprotein [155,187]<br>Ceruloplasmin [155,187]<br>α_1-antichymotrypsin [155,187]<br>α_1B-glycoprotein [155,187]<br>TSH and TF_4 [4,188]<br>β-trace protein [180,189]
**Dietery therapy**	Gal suppl [65,208,209]<br>Uridine suppl [209,210]<br>Glucose IV administration [211]	GlcNAc Suppl [67]	Ketogenic diet [216]	Sodium Phenylbutyrate suppl [57]	Vitamine B6 suppl [220]	Man (Man-1-P) suppl http://glycomine.com/ [46,47,52,77,194,202]<br>Glc starvation [194]

Supplementary Table 10 - continued.

	PGM1-CDG Phosphogluco-mutase 1	PGM3-CDG Phosphogluco-mutase 3	PIGA-CDG Phosphatidylinositol N-acetylglucosaminyl-transferase (subunit A)	PIGM-CDG GPI α-1,4-mannosyltransferase I	PIGO-CDG GPI ethanolamine phosphate transferase 3	PMM2-CDG Phosphomanno-mutase 2
**Transplantation**	Heart transplantation [65]	Hematopoietic cell transplantation [42]	NA	NA	NA	NA
**Gene therapy**	NA	NA	NA	NA	NA	NA
**Antisense therapy**	NA	NA	NA	NA	NA	AMO (c.640-15479C>T) [239]
**PCs**	NA	NA	NA	NA	NA	1-(3-chlorophenyl)-3-3-bis(pyridine-2-yl)urea [231]
**Others**	NPCRS [255,256] TPCRS [260]	NPCRS [255,256]	NPCRS [255,256]	NPCRS [255,256]	NPCRS [255,256]	Metformin [200]<br>MPI inhibitors [59,237]<br>NPCRS [255–258] ICARS [257,258]

Supplementary Table 11 - continued.

	**SLC35A1-CDG CMP-sialic acid transporter**	**SLC35A2-CDG UDP-galactose transporter**	**SLC35C1-CDG GDP-fucose transporter**	**SLC39A8-CDG Solute carrier family 39 (zinc transporter), member 8**	**SRD5A3-CDG Steroid 5α-reductase type 3**	**TMEM165-CDG Transmembrane protein 165**
**CTs**	NA	NA	NA	Interventional NCT02955264 Mn^{2+} trial (registered)	Interventional NCT02955264	Interventional NCT02955264
***In vitro* models**	CHO *Lec 2* mutants [17,25,26,38,125]<br>CHO MAR-11 mutant [126]<br>HAP1 *Slc35a1* knockout [32]	CHO *Lec8* mutant [33]<br>CHO *Lec8* mutant [33]	CHO *Slc35c1* knockout [130]<br>CHO *Slc35c1* knockout derived from MAR-11 mutants [128,129]<br>ESC *Slc35c1* knockout [131]	NA	*S.cerevisiae Dfg10-100* [140]	*S. cerevisiae Gdt1Δ* [30,141,142]<br>HEK293 *TMEM165* knockout and knockdown [36,142]<br>HeLa *TMEM165* knockdown [142]
***In vivo* models**	NA	*C. elegans Srf-1* mutants [127]	Mouse (*$Slc35c1^{-/-}$*) [132,133]<br>Zebrafish (*slytherin*) [135,136]<br>Zebrafish (Slc35c1 overexpression) [137]	Mouse ($Slc39a8^{(neo/neo)}$) [138,139]<br>Mouse ($Slc39a8^{-/-}$) [85]	Mouse (*$Srd5a3^{Gt/Gt}$*) [140]	Zebrafish (*$tmem165^{-/-}$*) [143]
**Biomarkers**	NA	NA	NA	NA	NA	NA

Supplementary Table 12 - continued.

	**SLC35A1-CDG CMP-sialic acid transporter**	**SLC35A2-CDG UDP-galactose transporter**	**SLC35C1-CDG GDP-fucose transporter**	**SLC39A8-CDG Solute carrier family 39 (zinc transporter), member 8**	**SRD5A3-CDG Steroid 5α-reductase type 3**	**TMEM165-CDG Transmembrane protein 165**
**Diatery therapy**	SA, ManNAc or the fetuin suppl (all w/o beneficial effects) [71]	Gal suppl [33,221]	Fuc suppl [23,27,50,51,53,132,136,222]	Mn^{2+} suppl [226,228]<br>Uridine suppl [226,227]<br>Gal suppl [226,227]	NA	Mn^{2+} suppl [70,142]<br>Gal suppl [70]
**Transplantation**	NA	NA	NA	NA	NA	NA
**Gene therapy**	NA	NA	NA	NA	NA	NA
**Antisense therapy**	NA	NA	NA	NA	NA	AMO (c.792+182G>A) [238]
**PCs**	NA	NA	NA	NA	NA	NA

Supplementary Table 13 – continued.

	**SLC35A1-CDG CMP-sialic acid transporter**	**SLC35A2-CDG UDP-galactose transporter**	**SLC35C1-CDG GDP-fucose transporter**	**SLC39A8-CDG Solute carrier family 39 (zinc transporter), member 8**	**SRD5A3-CDG Steroid 5α-reductase type 3**	**TMEM165-CDG Transmembrane protein 165**
**Others**	NPCRS [255,256]	NPCRS [255,256]	NPCRS [255,256]	NPCRS [255,256]	NPCRS [255,256]	NPCRS [255,256]

Supplementary Table 14 – continued.

	**TMEM199-CDG Transmembrane protein 199**						
**CTs**	NA	***In vivo* models**	NA	**Antisense therapy**	NA	**PCs**	NA
***In vitro* models**	DJY62/DJY102 (pep4–3 vma12 Δ::LEU2) DJY63 (vma12 Δ::LEU2) [105,144]	**Trans-plantation**	NA	**Biomarkers**	NA	**Others**	NPCRS [255,256]
	HeLa cell line (TMEM199 knowdown by CRISPR/Cas9) [106]	**Gene therapy**	NA	**Diatery therapy**	Iron supp [106]		

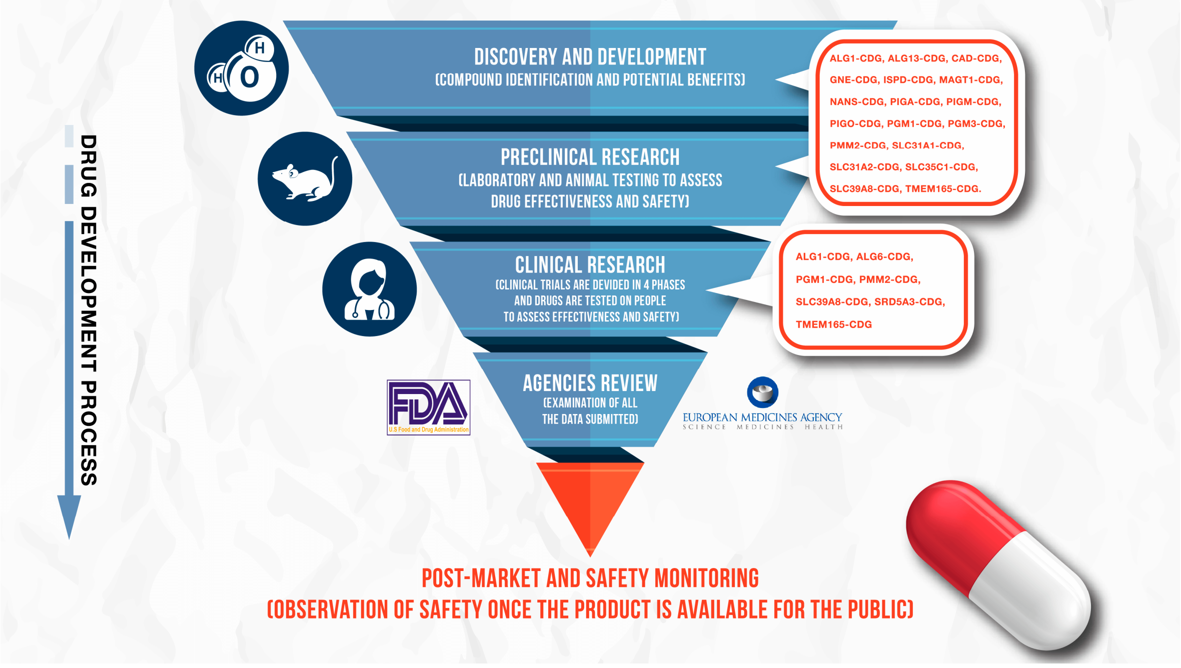

Figure S1 – Drug development process overview.

Animal models	Liver transplant	Heart transplantation	N-acetyl-D-mannosamine (ManNAC)
Yeast	Heart transplant	Transplantation	Mannosamine (ManN)
Drosophila melanogaster	Bone marrow transplant	Stem cell transplantation	Ribitol
Zebrafish	Transplant	Oral supplementation	Magnesium (Mg2+)
Mouse	Pharmacological chaperones	Mannose	N-Acetyl glucosamine (GlcNAc)
Rat	Chaperones	Galactose	Phenylbutyrate
Clinical trials	Antisense therapy	Fucose	Guanosine diphosphate
Therapies	Man-1-P	Sialic Acid	Uridine-5'-Diphosphate
C.elegans	Man-1-P therapy	Manganese	L-Aspartic acid
Therapy	Splicing	Uridine	L-Glutamine
Therapeutic strategies	Induced pluripotent stem cells	Metformin	Famotidine
Therapeutic options	Biomarkers	Acetazolamide	Phosphoric acid

IN COMBINATION WITH

Congenital disorders of glycosylation	ALG1	ALG1-CDG	ALG6
ALG6-CDG	ALG12	ALG12-CDG	ALG13
ALG13-CDG	ATP6VAP1	ATP6VAP1-CDG	CAD
Carbohydrate deficient glycoprotein syndrome	CAD-CDG	CCDC115	CCDC115-CDG
COG5	COG5-CDG	DOLK	DOLK-CDG
GNE	GNE-CDG	ISPD	ISPD-CDG
MAGT1	MAGT1-CDG	MPI	MPI-CDG
NANS	NANS-CDG	PGM1	PGM1-CDG
PGM3	PGM3-CDG	PIGM	PIGM-CDG
PMM2	PMM2-CDG	SL39A8	SL39A8-CDG
SLC35A2	SLC35A2-CDG	SLC35C1	SLC35C1-CDG
SRD5A3	SRD5A3-CDG	TMEM165	TMEM165-CDG
PIGA	PIGA-CDG	PIGO	PIGO-CDG

Figure S2 – List of all keywords used for this systematic literature review.

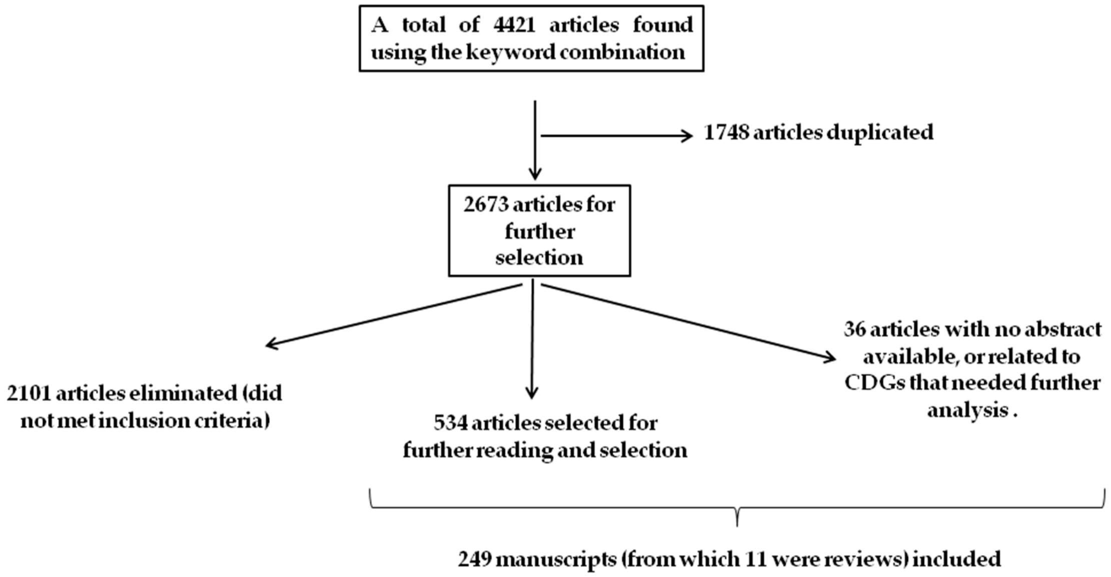

Figure S3 – Diagram of the inclusion/elimination process used for manuscript selection.
